# Repeated exposure to heterologous hepatitis C viruses associates with enhanced neutralizing antibody breadth and potency

**DOI:** 10.1172/JCI160058

**Published:** 2022-08-01

**Authors:** Nicole Frumento, Alexis Figueroa, Tingchang Wang, Muhammad N. Zahid, Shuyi Wang, Guido Massaccesi, Georgia Stavrakis, James E. Crowe, Andrew I. Flyak, Hongkai Ji, Stuart C. Ray, George M. Shaw, Andrea L. Cox, Justin R. Bailey

**Affiliations:** 1 Department of Medicine and; 2 Department of Biostatistics, Johns Hopkins University Bloomberg School of Public Health, Baltimore, Maryland, USA.; 3 University of Bahrain, Department of Biology, College of Science, Sakhir Campus, Bahrain.; 4 Department of Medicine and; 5 Department of Microbiology, University of Pennsylvania, Philadelphia, Pennsylvania, USA.; 6 Department of Pathology, Microbiology and Immunology,; 7 Department of Pediatrics, and; 8 Vanderbilt Vaccine Center, Vanderbilt University Medical Center, Nashville, Tennessee, USA.; 9 Division of Biology and Biological Engineering, California Institute of Technology, Pasadena, California, USA.

**Keywords:** Immunology, Virology, Adaptive immunity, Antigen, Hepatitis

## Abstract

A prophylactic hepatitis C virus (HCV) vaccine that elicits neutralizing antibodies could be key to HCV eradication. However, the genetic and antigenic properties of HCV envelope (E1E2) proteins capable of inducing anti-HCV broadly neutralizing antibodies (bNAbs) in humans have not been defined. Here, we investigated the development of bNAbs in longitudinal plasma of HCV-infected persons with persistent infection or spontaneous clearance of multiple reinfections. By measuring plasma antibody neutralization of a heterologous virus panel, we found that the breadth and potency of the antibody response increased upon exposure to multiple genetically distinct infections and with longer duration of viremia. Greater genetic divergence between infecting strains was not associated with enhanced neutralizing breadth. Rather, repeated exposure to antigenically related, antibody-sensitive E1E2s was associated with potent bNAb induction. These data reveal that a prime-boost vaccine strategy with genetically distinct, antibody-sensitive viruses is a promising approach to inducing potent bNAbs in humans.

## Graphical abstract

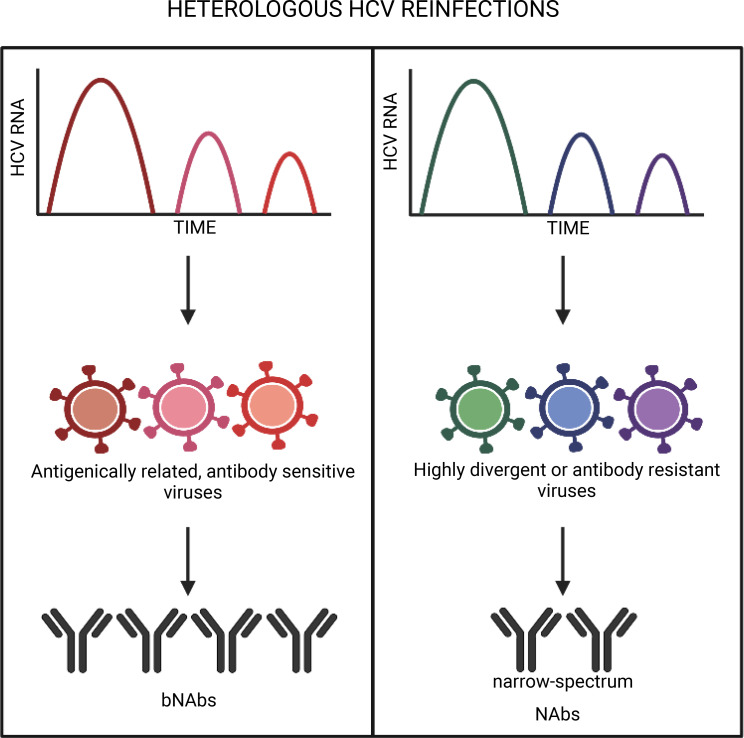

## Introduction

An estimated 71 million people are infected with hepatitis C virus (HCV) worldwide (1). Given the largely asymptomatic nature of this disease, only a fraction of the HCV-infected population is aware that they are infected (2). Of the individuals who are diagnosed, only a small percentage are effectively treated with direct-acting antiviral (DAA) therapy. Although treatment is effective and safe, it remains unavailable to many, especially in resource-limited settings, and curative treatment does not prevent reinfection (3). Persons with chronic HCV infection are at risk for complications, such as cirrhosis, end-stage liver disease, and hepatocellular carcinoma, and cirrhosis-related risks persist even after HCV cure (4, 5). Therefore, an HCV vaccine is needed to achieve disease eradication (6, 7).

One of the major challenges to the development of a successful HCV vaccine is the extraordinary genetic diversity of the virus (8–10). Fortunately, multiple broadly neutralizing Abs (bNAbs) have been identified that block infection by diverse HCV strains in vitro, and infusion of bNAbs is protective against HCV infection in animal models (11–17). In humans, along with potent antiviral T cell responses, early development of plasma bNAbs has been associated with spontaneous clearance of primary HCV infection, which occurs in about 25% of infected individuals (18–20). Notably, these immune responses do not provide sterilizing immunity, since individuals can be reinfected after HCV clearance. However, about 80% of those who clear their first infection clear subsequent reinfections (21). Reinfections are associated with a rapid rise in neutralizing Ab (NAb) titers, shorter duration of infection, and lower peak viremia, which indicate protection by adaptive immunity (21–23). Thus, individuals who clear multiple reinfections can serve as a model for a desired vaccine response.

However, questions remain about how anti-HCV bNAbs are induced in humans. It remains unclear whether multivalent and/or prime-boost immunizations are needed to induce anti-HCV bNAbs and what genetic or antigenic criteria should be used to select vaccine antigens. There is evidence that sequential exposure to dengue, influenza, or HIV antigens can lead to broader immune responses (24–26). For chronically HCV-infected individuals, longer duration of infection leads to greater neutralizing breadth (19, 27–29). While reinfection after HCV clearance can lead to broadening of the NAb response (27), not all reinfected individuals develop bNAbs, and the genetic or antigenic features of primary and reinfecting viruses associated with acquisition of bNAbs have not been defined. For example, it is unclear whether exposure to multiple highly diverse variants or reinfection with antigenically similar viruses is required to induce a potent bNAb response. Characterization of these genetic and antigenic features can inform development of a prophylactic HCV vaccine.

**Figure 1:**
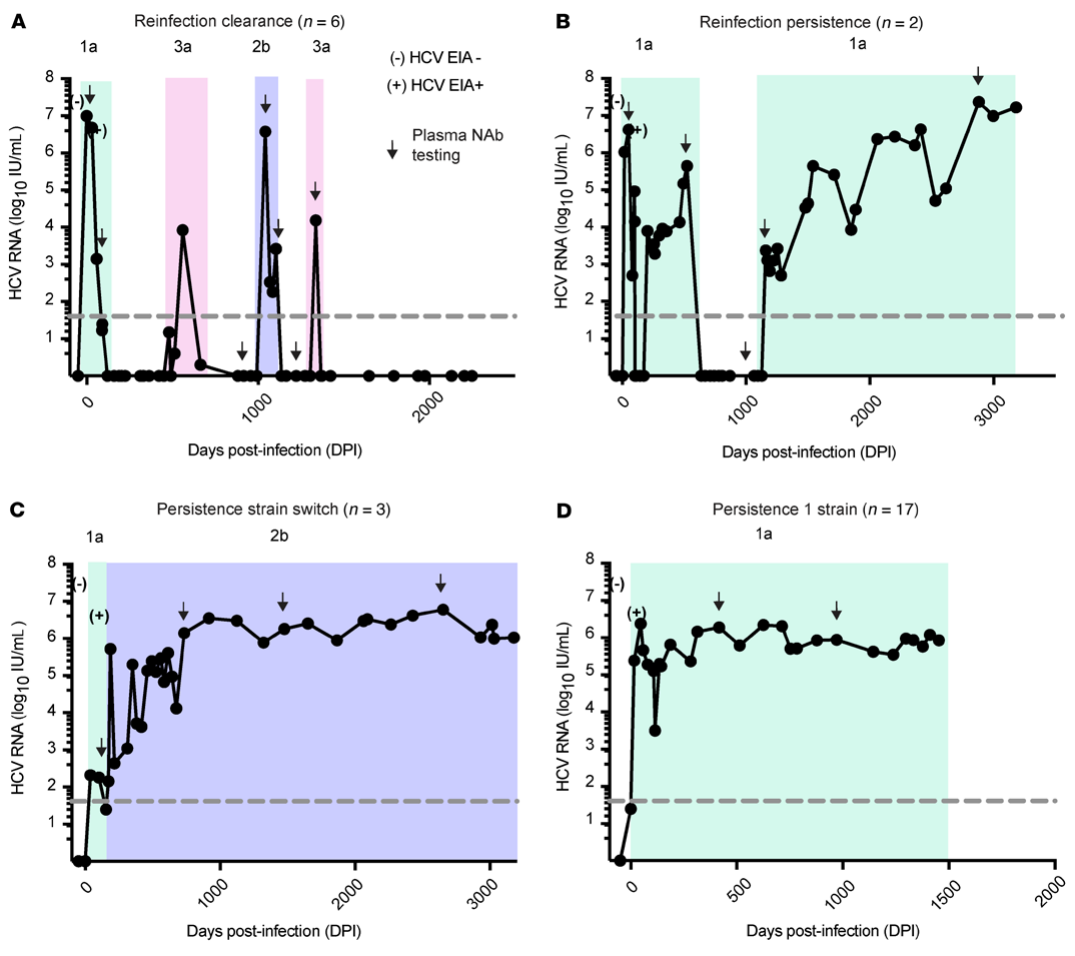
Representative graphs demonstrating the history of viremia of study participants. Representative viral load graphs of HCV-infected participants with (**A**) cleared reinfection, (**B**) persistent reinfection, (**C**) persistent infection with a strain switch, or (**D**) persistent infection with 1 strain. The study was designed for monthly viral load testing, with more than 8 years of follow-up in some individuals. Dashed line indicates limit of detection (LOD) of the HCV RNA assay. Infections are shaded with different colors based on the HCV subtype of the infecting virus, with subtype indicated, determined by sequencing of Core-E1 genes.

In this study, we aimed to define the antigenic stimuli that drive the development of potent anti-HCV bNAbs in humans. We assessed development of bNAbs in a prospective, longitudinal cohort of persons who inject drugs (PWIDs) who acquired HCV infection during follow-up, including study participants with (a) spontaneous clearance of primary infection and multiple reinfections, (b) clearance of primary infection followed by persistent reinfection, (c) persistent, sequential infections with genetically distinct viruses, or (d) persistent, chronic infection with a single viral strain (Figure 1). We measured neutralizing breadth and potency of plasma Abs at multiple time points in each study subject and identified the mAb types responsible for the neutralizing activity of each plasma sample. We evaluated the relationship between development of bNAbs and (a) exposure to multiple genetically distinct infections, (b) duration of viremia, (c) genetic distance between primary infection and reinfection viruses, and (d) antigenic similarity between primary infection and reinfection viruses. We used these data to develop a rigorous model identifying key features of stimuli capable of inducing potent bNAbs in humans.

## Results

### Selection of study participants.

Study participants were PWIDs enrolled in the Baltimore Before and After Acute Study of Hepatitis (BBAASH) cohort who were identified before or very early during acute HCV infection (prior to HCV Ab seroconversion) and subsequently followed in a study designed for monthly follow-up through spontaneous clearance of primary HCV infections and reinfections or over years of chronic infection. Study participants were divided into 4 groups based on different patterns of infection (Figure 1). Reinfection clearance subjects (*n =* 6) were defined as individuals who were infected with and subsequently cleared without treatment multiple infections with genetically distinct HCV strains (divergence between strains ≥0.03), with an interval of at least 60 days of aviremia between infections (Figure 1A). Reinfection persistence subjects (*n =* 2) were defined as HCV-infected persons who cleared their first infection without treatment and, after an interval of at least 60 days of aviremia, were subsequently reinfected with another genetically distinct virus that was not cleared (Figure 1B). Persistence strain switch subjects (*n =* 3) were infected sequentially with more than one genetically distinct viral strain without a detected interval of aviremia between the first and second infections (Figure 1C). For these subjects, we considered the first infection prior to the viral strain switch the primary infection and subsequent infection after the viral strain switch as a reinfection. Finally, as controls for this study, we selected participants who remained HCV infected with a single HCV strain (divergence between longitudinal viruses <0.03) over many years of follow-up (designated persistence 1 strain subjects, *n =* 17; Figure 1D). Overall, the subject age at the time of seroconversion, sex, race, and HCV infection genotype were not significantly different between the groups (Table 1).

### Plasma neutralization of a heterologous virus panel.

**Figure 2:**
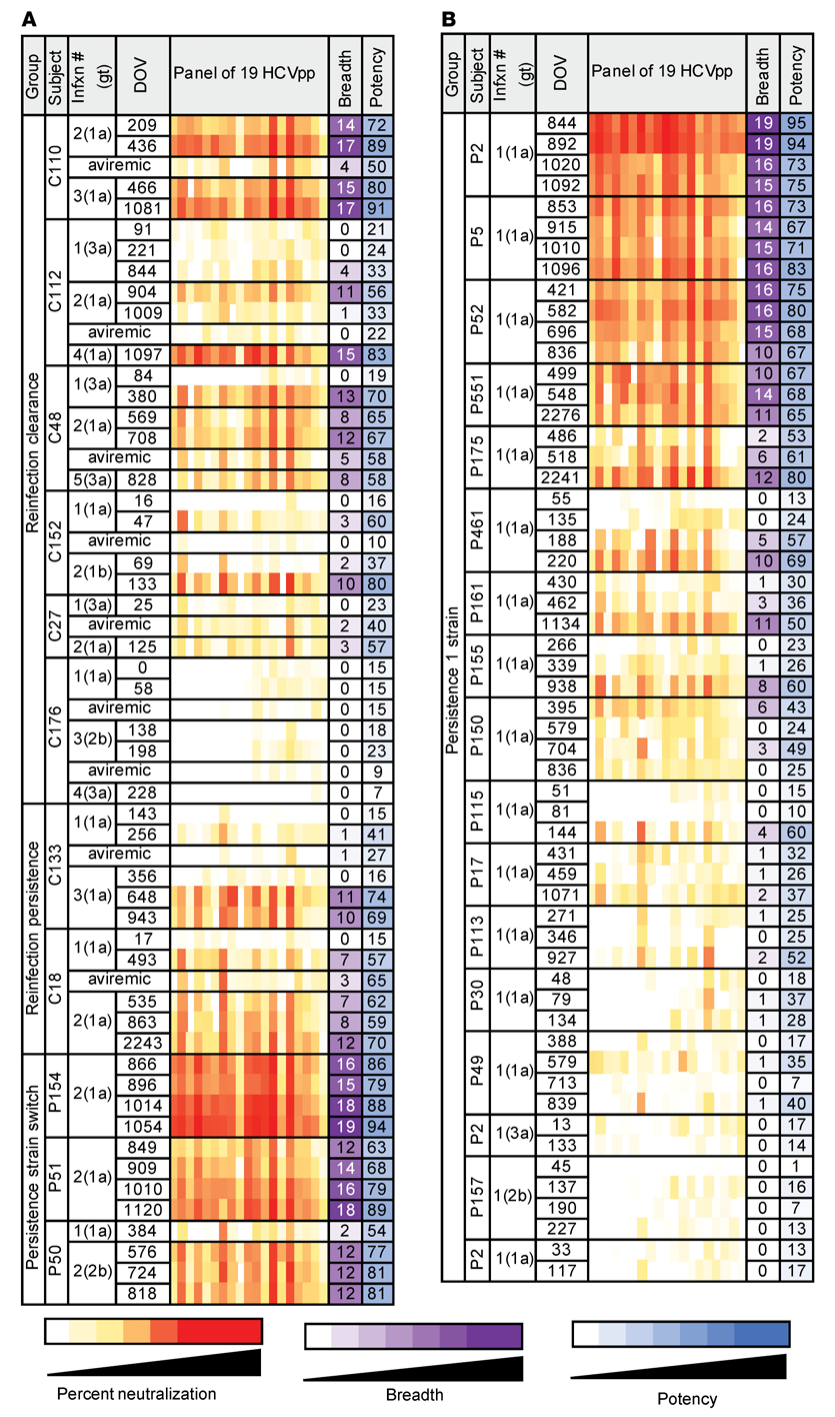
Neutralizing breadth and potency of Abs in longitudinal plasma samples. (**A**) Percentage neutralization of 19 HCVpp by plasma of (**A**) reinfection clearance, reinfection persistence, persistence strain switch subjects, and (**B**) persistence 1 strain subjects. Subjects from each group are arranged from highest to lowest neutralizing breadth. Negative percentage neutralization values were converted to 0. DOV, calculated by counting viremic periods and excluding periods of aviremia between infections; Infxn # (gt), number of genetically distinct infections the subject has experienced (genotype of the current infection); breadth, number of HCVpp neutralized at least 25% by plasma at 1:100 dilution; potency, highest percentage of neutralization across the panel of 19 HCVpp by plasma at 1:100 dilution. Percentage neutralization values are the average of 2 independent experiments performed in duplicate.

We measured neutralizing activity of Abs in participants’ plasma obtained at longitudinal time points during infection using a panel of 19 genotype 1a and 1b HCV pseudoparticles (HCVpp). This panel comprises HCVpp with a range of neutralization sensitivity expressing 94% of the amino acid polymorphisms present at greater than 5% frequency in a reference panel of 643 genotype 1 HCV isolates from GenBank (19). The neutralizing breadth of mAbs measured with this genotype 1 panel and neutralizing breadth of the same mAbs measured with HCV strains from genotypes 1 to 6 was shown to be similar in previous studies (30–32). Furthermore, we have observed a strong correlation between breadth of plasma from HCV-infected individuals measured with this panel of 19 genotype 1 HCVpp and breadth of the same plasma measured with an antigenically diverse panel discovered more recently that includes multiple genotypes (Supplemental Figure 1; supplemental material available online with this article; https://doi.org/10.1172/JCI160058DS1) (19, 33). We tested plasma samples collected at or immediately after the first viremic time point, prior to clearance of primary infection, prior to reinfection, immediately after reinfection, and prior to clearance of reinfection (reinfection clearance group) or at days 587–1708 of reinfection (reinfection persistence group) (Figure 1). We also tested samples from persistence 1 strain and persistence strain switch subjects that were time matched with reinfection subjects based on days of viremia (DOV) (Figure 2, A and B). We defined plasma Ab–neutralizing breadth as the number of HCVpp neutralized more than 25% by a 1:100 dilution of the plasma and potency as the highest percentage neutralization value across the panel of 19 HCVpp by the same dilution of plasma. We selected this 25% neutralization cutoff based upon mean and standard deviation of neutralization by a negative control isotype mAb (33). Close to 60% of subjects showed an increase in plasma-neutralizing breadth and potency over the course of primary infection and reinfection, regardless of the outcome of infection, while the rest showed very low or no plasma Ab neutralization of the HCVpp panel (Figure 2). Overall, there was a nonsignificant trend toward higher median breadth and potency for clearance samples compared with time-matched persistence samples (Supplemental Figure 2, A and B). Similarly, when the analysis was restricted to individuals with detectable plasma-neutralizing activity (capable of neutralizing at least 1 HCVpp), there was no significant difference in frequency of individuals with detectable neutralizing activity or in median breadth between the clearance and persistence groups (Supplemental Figure 2, C and D). Lack of statistical significance may be due to the small sample size of the groups. Persistence strain switch subjects had the highest median breadth and potency of all groups. This finding was true both when comparing overall breadth and potency of all time-matched plasma samples and highest breadth and potency for each subject (Table 1).

### Deconvolution of NAb types in plasma of HCV-infected subjects.

**Figure 3:**
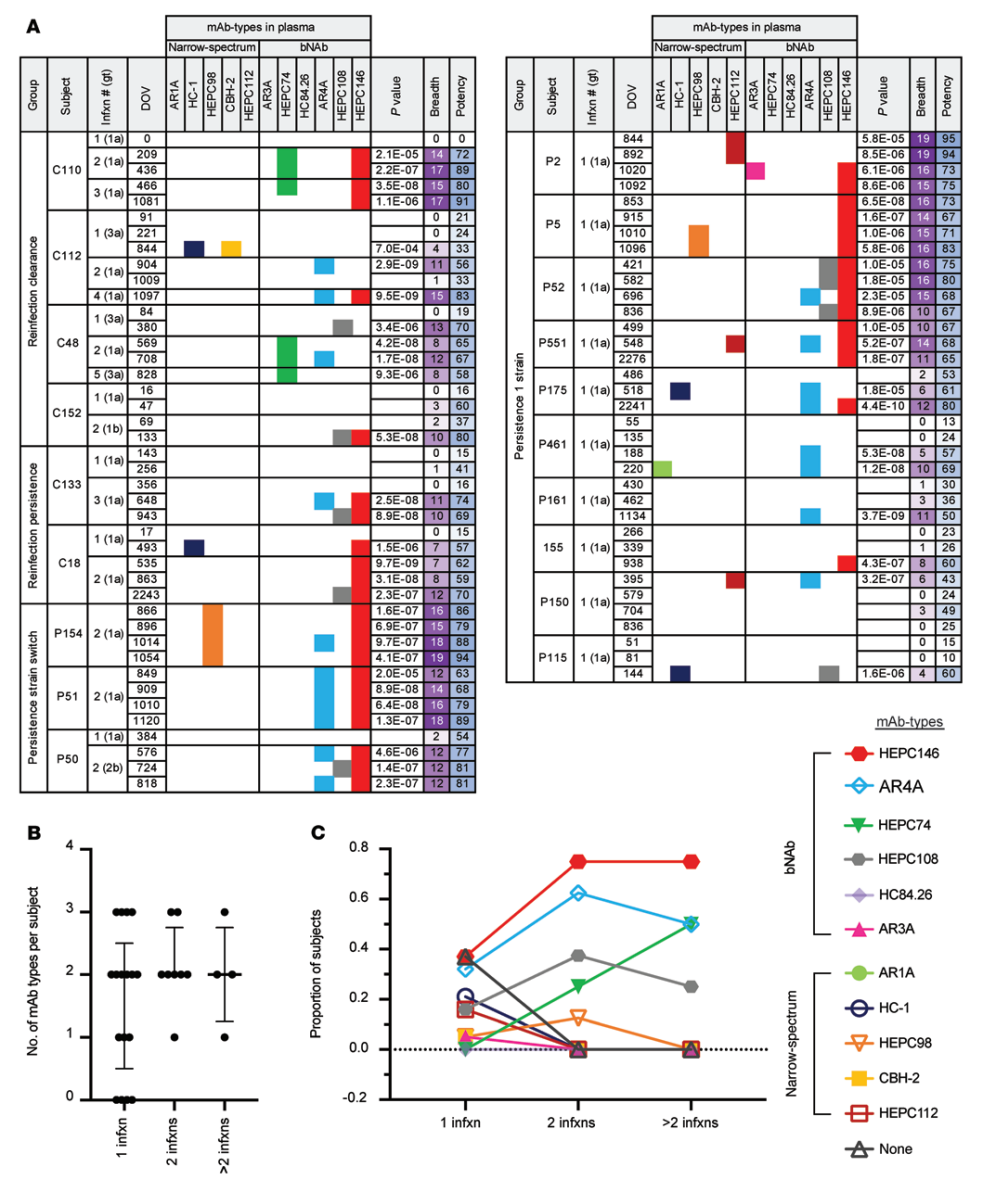
Plasma deconvolution reveals mAb types contributing to plasma-neutralizing breadth and potency. (**A**) mAb types identified by deconvolution of neutralizing activity of each plasma sample. Neutralization profiles entered into the deconvolution algorithm were averaged from 2 independent neutralization experiments performed in duplicate. For each plasma sample, mAb types with deconvolution values exceeding the true positive cutoff are indicated, with a different color assigned to each mAb type. Reference mAbs were designated narrow spectrum or bNAbs based on neutralization of less than 50% or more than 50% of the HCVpp panel. Deconvolution was performed only for plasma samples with neutralizing breadth greater than or equal to 4. This breadth 4 or greater cutoff was determined using control mAb “spike-in” experiments to determine the minimum neutralizing activity necessary for accurate Ab deconvolution. Subjects with neutralizing breadth of less than 4 for all samples were excluded from this analysis. Plasma samples are grouped by subject outcome. *P* values were calculated for Pearson’s correlation between the plasma sample neutralization profile and the best fit combined reference mAb neutralization profile. Breadth, number of HCVpp neutralized at least 25% by plasma at 1:100 dilution; potency, highest percentage neutralization across the panel of 19 HCVpp by plasma at 1:100 dilution. (**B**) Number of mAb types detected per subject after 1 infection (*n =* 17), after 2 infections (*n =* 8), and after more than 2 infections (*n =* 4). Median with IQR is shown. (**C**) The proportion of subjects with each mAb type (or 0 mAb-types) after 1 infection, after 2 infections, and after more than 2 infections (*n =* 4).

We applied a plasma NAb deconvolution algorithm to identify mAb types responsible for the plasma-neutralizing breadth and potency observed for each sample (Figure 3A) (34). We generated a neutralization profile for each plasma sample with breadth equal to or greater than 4 HCVpp by ranking its relative neutralization potency across the 19 HCVpp in the panel. By comparing these plasma neutralization profiles to the neutralization profiles of a panel of 11 HCV E1E2-specific reference mAbs using code in R, we deconvoluted the combination of mAb types present in each plasma sample. The reference panel included mAbs targeting neutralizing epitopes across E1 and E2 (AR1, AR3, AR4, HVR1, domain B, domain C, domain D), and we improved the previously described method by expanding the panel of reference mAbs to include mAbs targeting 3 additional distinct antigenic sites: HEPC108 (a bNAb that binds the E2 central β sheet and front layer), HEPC146 (a bNAb that binds the E2 CD81-binding loop), and HEPC112 (a NAb that binds E1) (31). Using deconvolution of control samples with spiked-in mAbs, we determined the true positive threshold for identification of each reference mAb in monoclonal or polyclonal mixtures (Supplemental Table 1). A mAb type was called positive in test plasma samples only if its deconvolution value exceeded this threshold. To validate the plasma deconvolution method after the addition of the new reference mAbs, we performed deconvolution analysis on plasma of human subjects C110 and C18, from whom we had previously isolated E1E2-specific mAbs from peripheral blood B cells (30, 31). As expected, HEPC74-type NAbs were identified in plasma of C110, the source of HEPC74-producing B cells. HEPC108-type and HEPC146-type NAbs were each identified in plasma of C18, the source of HEPC108- and HEPC146-producing B cells (Supplemental Figure 4).

Deconvolution demonstrated that plasma-neutralizing breadth and potency observed in the study participants could be attributed to a variety of mAb types in plasma (Figure 3A). We detected a median of 2 mAb types per subject during primary infection or subsequent reinfections (Figure 3B). Notably, we detected a larger variety of mAb types during primary infection, including some mAb types with narrow neutralizing breadth. However, by the second or later infection of most subjects, only HEPC146-, AR4A-, HEPC74-, and HEPC108-like responses were detected (Figure 3C). Taken together, these data show that some narrow-spectrum mAb types were present in plasma during primary infections, but these responses were superseded by 4 bNAb-types that became dominant during reinfections.

### Duration of viremia and number of distinct infections are associated with greater plasma-neutralizing breadth and potency regardless of infection outcome.

**Figure 4:**
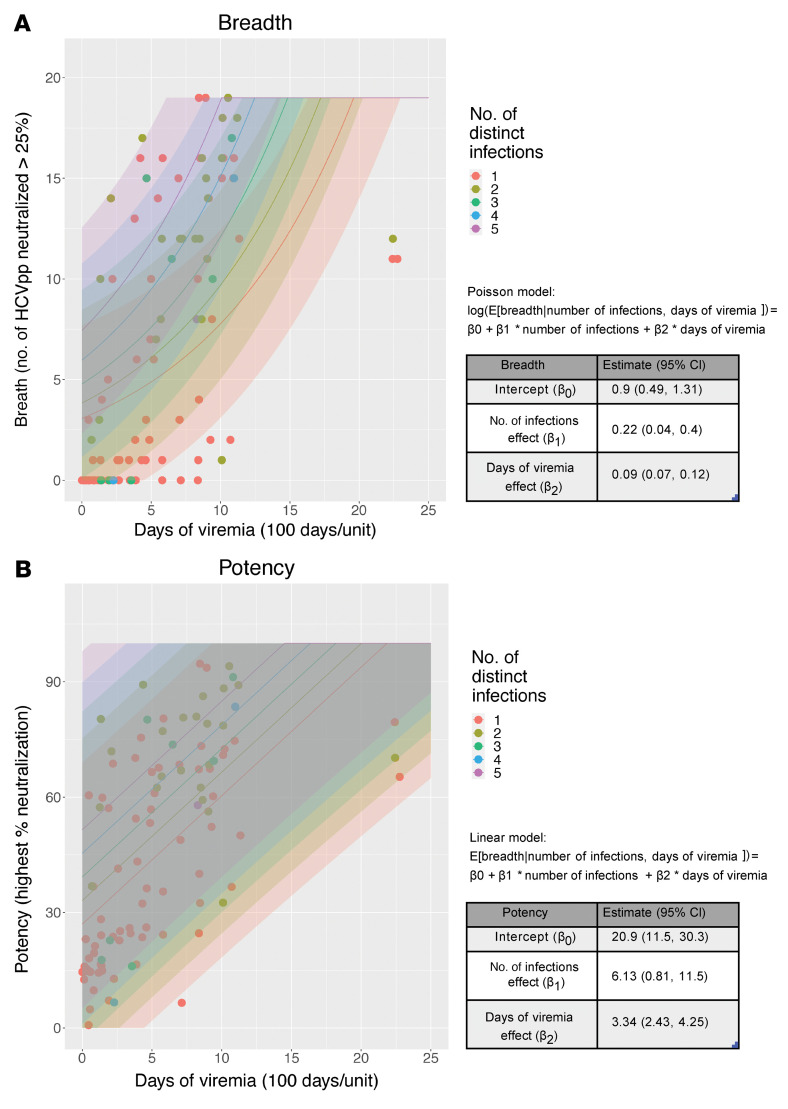
Duration of viremia and number of distinct infections are associated with increased neutralizing breadth and potency. (**A**) Quasi-Poisson regression analysis for the association of number of infections and duration of infection with neutralizing breadth. Curve and 95% prediction intervals (shaded areas) for each number of infections are indicated with different colors. The regression model equation and table with the estimated coefficients and 95% CI for each variable are shown (right). (**B**) Linear regression analysis for the association of number of infections and duration of infection with neutralizing potency. Curves and 95% prediction intervals (shaded areas) for each number of infections are indicated with different colors. The linear model equation and table with the estimated coefficients and 95% CI for each variable are shown (right).

To identify variables associated with greater plasma-neutralizing breadth, we performed regression analysis to model the effect on neutralizing breadth of duration of viremia, number of distinct infections (infections with genetic nucleotide distance ≥0.03), and outcome of infection. We divided the data set into 2 subsets containing information from persistent or cleared infections. The DOV variable was scaled and measured in 100 days per unit. Since neutralizing breadth is an integer count, we conducted the analysis using Poisson regression with quasi likelihood to account for overdispersion (i.e., quasi-Poisson). The residuals from the model are well scattered around zero, suggesting good model fitting (Supplemental Figure 5A). We examined the necessity of inclusion of outcome of infection by conducting an *F* test between 2 nested models with or without this variable. Since the 2 models were not significantly different (*P =* 0.41), the outcome of infection variable was subsequently excluded from the regression model for breadth. This finding indicates that clearance or persistence of infection was not significantly associated with breadth in our analysis. Instead, the quasi-Poisson regression determined that neutralizing breadth was significantly associated with days of viremia and number of distinct infections per subject (95% CI of the regression coefficient does not cross 0 for either variable) (Figure 4A).

Similar results were observed when modeling the effect of duration of viremia, number of distinct infections, and outcome of infection on plasma-neutralizing potency (Figure 4B). Since neutralizing potency is a continuous variable, a linear regression model was used. The residuals were scattered and well spread, indicating good model fitting (Supplemental Figure 5B). Here too, the models with or without the outcome of infection variable were not significantly different from one another (*P =* 0.98). As with neutralizing breadth, neutralization potency was significantly associated with days of viremia and number of distinct infections per subject (95% CI of the regression coefficient does not cross 0 for either variable) (Figure 4B). Therefore, duration of viremia and number of distinct infections are associated with greater plasma-neutralizing breadth and potency in HCV infection regardless of whether the infection is cleared or persists.

### Greater genetic distance between infecting viruses is not associated with greater plasma-neutralizing breadth or potency.

**Figure 5:**
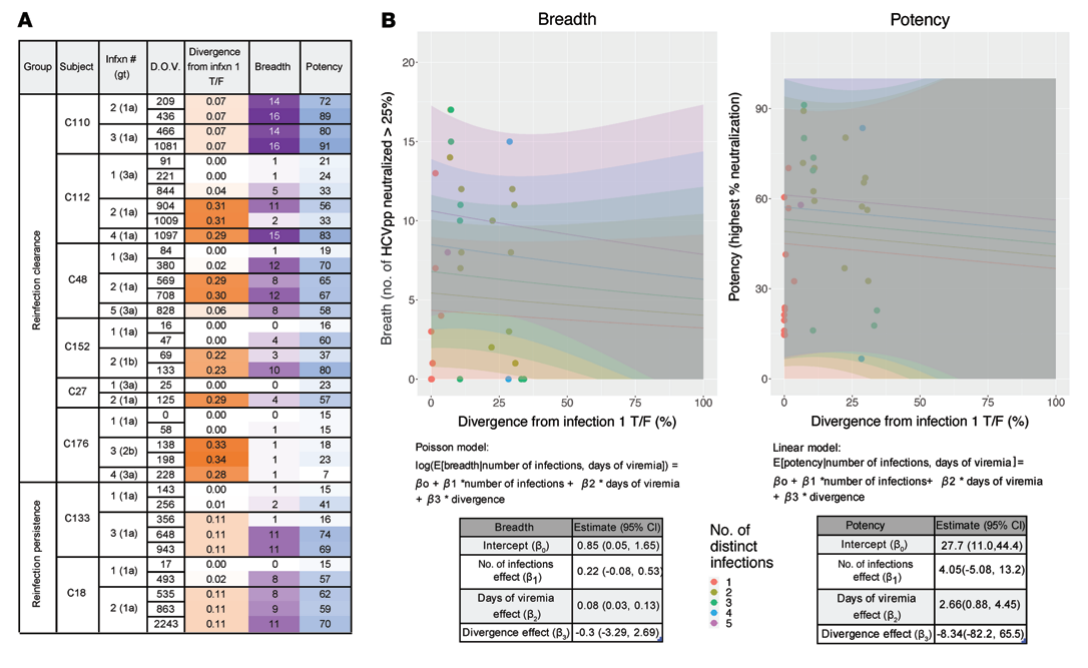
Greater genetic distance between infecting viruses is not associated with increased neutralizing breadth or potency. (**A**) Table illustrating divergence of each reinfecting virus E1E2 sequence (amino acid *p* distance) from that subject’s infection 1 T/F virus E1E2 sequence. (**B**) Quasi-Poisson regression (left) and linear regression (right) analyses for the association of divergence, number of infections, and DOV with neutralizing breadth (left) or neutralizing potency (right). Curves and 95% prediction intervals (shaded areas) for each number of infections are shown in different colors. Model equations and tables with the estimated coefficients and 95% CI for each variable are shown (below).

Next, we evaluated whether greater genetic distance between primary infection and reinfection viruses was also associated with greater plasma-neutralizing breadth or potency. First, we compared plasma breadth and potency during reinfections with the same or different HCV subtype as the primary infection and we observed that they were not significantly different from one another (Supplemental Figure 6). We then treated genetic distance as a continuous variable by measuring divergence of reinfection E1E2 sequences from primary infection transmitted/founder (T/F) virus E1E2 of each subject (Figure 5A). We selected the most frequently observed primary infection T/F virus to use in this analysis (Supplemental Table 2). Divergence was determined by calculating the amino acid *p* distance between each reinfection E1E2 sequence and the most frequently observed primary infection T/F virus E1E2 sequence from the same subject. This analysis was limited to subjects from whom we had previously obtained 5′ hemigenome sequences by single-genome amplification (SGA) (*n =* 8). We then modeled the relationship among E1E2 divergence of the reinfecting viruses, duration of viremia, number of distinct infections, and neutralizing breadth. We compared the model with and without genetic divergence via *F* test and determined that inclusion of this variable did not significantly improve the model (*P =* 0.85). We then used the model including the genetic divergence variable to make predictions about breadth. Greater genetic divergence of each reinfecting virus from the primary infection T/F virus was not associated with greater neutralizing breadth in reinfection subjects, as illustrated by the fact that the 95% CI for the divergence regression coefficient in the quasi-Poisson model contains 0 (Figure 5B). Similarly, when we evaluated the effect of genetic divergence when predicting potency, the *F* test was not significant (*P =* 0.83) and the 95% CI for the divergence regression coefficient in the linear model covered 0 (Figure 5B). Therefore, greater genetic distance between primary and reinfecting viruses was not associated with greater neutralizing breadth or potency in reinfection subjects.

### Repeated infections with antigenically related, Ab-sensitive viruses were associated with greater plasma-neutralizing breadth and potency.

Since we found that repeated exposure to highly genetically divergent infecting viruses was not associated with higher breadth or potency, we analyzed the antigenicity of the infecting viruses. To do so, we measured binding in an ELISA of a panel of E1E2-specific mAbs to longitudinal E1E2 proteins generated from viruses of 8 of the study participants. This panel of mAbs included the mAbs used for neutralization profiling shown in Figure 3 along with the bNAb HC33.4 and the weakly neutralizing but broadly crossreactive mAb CBH-7. In addition, we hypothesized that E1E2 variants that were sensitive to binding of unmutated germline bNAb ancestors might play a role in early selection of bNAb-producing B cell lineages, so we also measured binding of inferred germline ancestors (recombinant unmutated ancestors [rua]) of bNAbs HEPC146, HEPC74, HEPC108, and HEPC98 (30).

To select the optimal mAb concentration for binding quantitation, we first performed binding curves with serial dilutions of a subset of the reference mAbs and a subset of E1E2 proteins (Supplemental Figure 7). From these curves, we identified a single mAb concentration (0.08 μg/mL) that fell in the exponential binding phase of most binding curves. Subsequent mAb-E1E2 binding tests were performed at this concentration. We tested rua mAbs at a higher concentration because they were known from prior experiments to have low binding affinity (35). The decision to test most mAb-E1E2 combinations at a single mAb concentration was validated by the high correlation between the EC_50_values and OD values for the mAb-E1E2 combinations that were tested both with full Ab titration curves and single mAb concentrations (*P <* 0.0001; Supplemental Figure 8).

**Figure 6:**
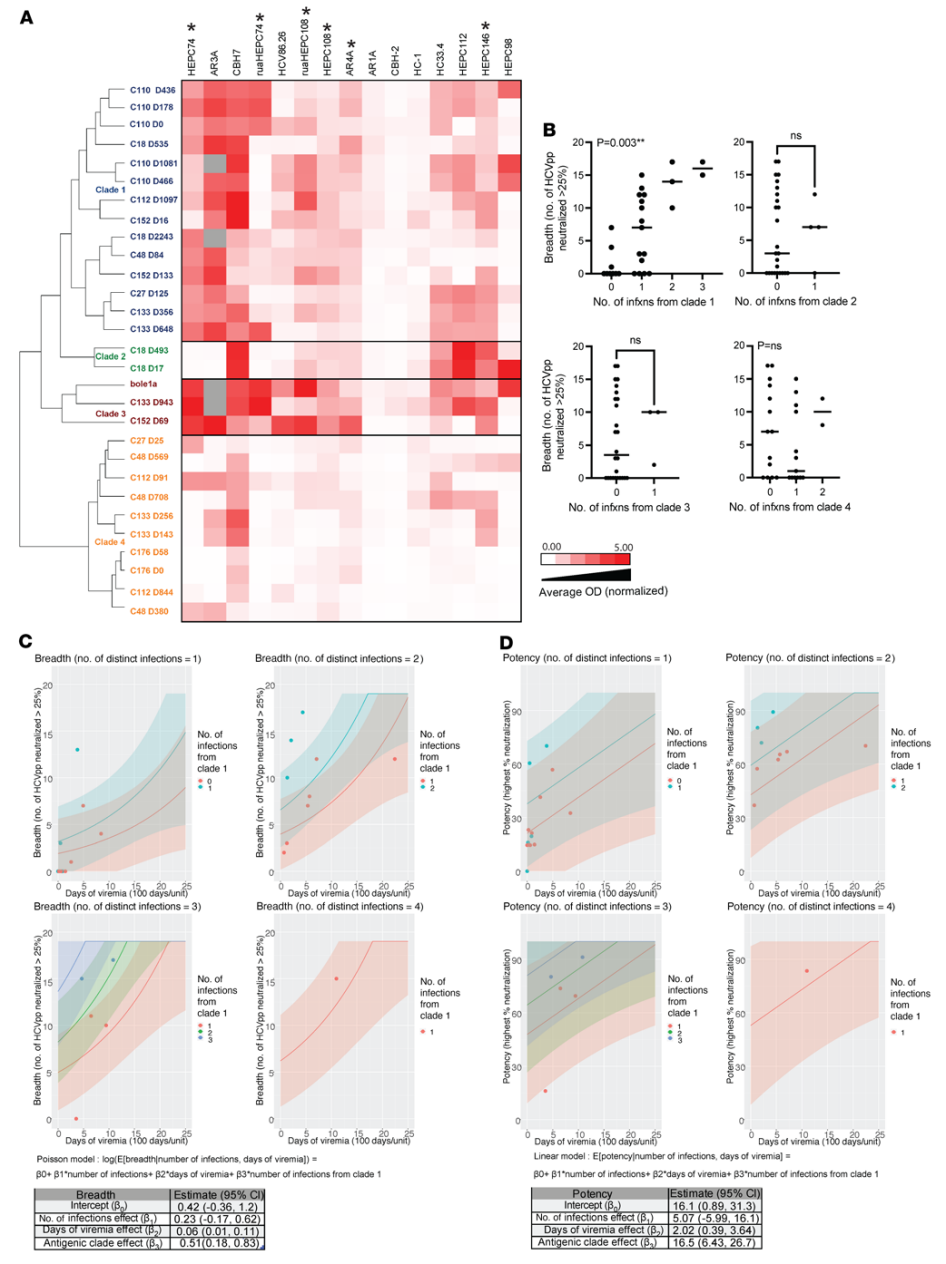
Longitudinal E1E2 isolates from reinfection subjects cluster in antigenically distinct clades, and infection with viruses from antigenic clade 1 is associated with increased neutralizing breadth and potency. (**A**) Heatmap illustrating ELISA binding of a panel of mAbs recognizing conformational epitopes to longitudinal E1E2 proteins from reinfection subjects. Each value is the average of 2 replicates and is normalized for binding of HCV-1, a control mAb recognizing a linear epitope that is 100% conserved across all isolates. Gray cells indicate missing data. E1E2 proteins were clustered based on mean squared distance between binding profiles. Asterisks indicate mAb types or ruas of mAb types identified in broadly neutralizing plasma after multiple infections (Figure 3). (**B**) Number of infections with viruses from antigenic clade 1 is significantly associated with greater neutralizing breadth, but number of infections with viruses from clades 2 through 4 are not (*P* > 0.05). Normality of data was tested by Shapiro-Wilk test. Kruskal-Wallis test (clades 1 and 4) and Mann-Whitney nonparametric test (clades 2 and 3) were conducted. Horizontal lines indicate medians. (**C** and **D**) Quasi-Poisson regression (**C**) and linear regression (**D**) analyses for the association of number of infections with viruses from antigenic clade 1, total number of infections, and DOV with neutralizing breadth (**C**) or neutralizing potency (**D**). Each total number of infections from 1 to 4 is illustrated on a separate graph. Curves and 95% prediction intervals (shaded areas) for each number of antigenic clade 1 infections are shown in different colors. Model equations and tables with the estimated coefficients and 95% CI for each variable are shown (below).

We measured binding of mAbs to longitudinal E1E2 proteins from reinfection subjects and to control E1E2 protein bole1a. We included bole1a, a computationally designed ancestral genotype 1a HCV sequence, because it is a genetically representative strain that we have previously demonstrated to be highly neutralization sensitive, with theoretical potential as a vaccine antigen (36–38). The E1E2s clustered into 4 major antigenic clades (designated clades 1–4) based on their patterns of relative binding by all reference mAbs (Figure 6A). Clade 1 included E1E2 proteins that were sensitive to most of the reference mAbs in the panel. Clade 3 included bole1a and 2 other E1E2 proteins from reinfection subjects, which were also relatively sensitive to binding of the reference mAbs. E1E2 proteins in clades 2 and 4 were resistant to binding of the majority of mAbs. All clades, except clade 2, contained E1E2 proteins from multiple genotypes and/or subtypes (Supplemental Figure 9), indicating that the antigenic characteristics of the proteins were not dictated by their genotypes.

We then explored the relationship between infections with viruses from the different antigenic clades, neutralizing breadth, and potency. At each time point, we counted the number of infections each subject had experienced with viruses from antigenic clades 1, 2, 3, or 4 as well as the number of infections with viruses from distinct antigenic clades, and we compared these values to the neutralizing breadth of plasma from the same time points (Supplemental Figure 10A). We found that only the number of distinct infections with viruses from antigenic clade 1 was significantly associated with greater neutralizing breadth (Figure 6B and Supplemental Figure 10B). We then modeled the relationship between number of infections with distinct viruses from antigenic clade 1, duration of viremia, total number of infections, and neutralizing breadth (Figure 6C). Although the total number of infections and the number of infections with viruses from antigenic clade 1 were highly correlated, inclusion of both variables was well tolerated by the model. The quasi-Poisson regression showed that the number of infections with antigenic clade 1 variants was highly associated with greater neutralizing breadth, as illustrated by the high regression coefficient with a 95% CI that did not cross 0. Similarly, a very high association between the number of infections with viruses expressing antigenic clade 1 E1E2 proteins and neutralizing potency was observed (Figure 6D). Notably, antigenic clade 1 viruses were particularly sensitive to binding of the bNAbs that were immunodominant in broadly neutralizing plasma of reinfected individuals (HEPC146, AR4A, HEPC74, and HEPC108; Figure 3) as well as germline precursors of 2 of those bNAbs (HEPC74rua and HEPC108rua). In conclusion, we found that repeated infections with antigenically related, Ab-sensitive viruses, together with longer duration of viremia, were significantly associated with greater neutralizing breadth and potency.

## Discussion

Selection of HCV vaccine antigens that effectively elicit Abs with strong neutralizing activity is critical. Here, we identified key features of the antigenic stimuli capable of inducing potent anti-HCV bNAbs in humans. We measured the neutralizing breadth and potency of Abs in longitudinal plasma of each study participant and identified 4 major bNAb types commonly induced upon reinfection. We showed that the neutralizing breadth and potency of the Ab response increased upon repeated exposure to genetically distinct HCV strains and with longer duration of viremia. We also found that a specific Ab-sensitive antigenic profile of the infecting strains, not greater genetic difference between strains, was associated with increased plasma breadth and potency in HCV-reinfected subjects.

Induction of bNAbs is a major goal of HCV vaccine development. To date, most candidate vaccines intended to induce bNAbs have relied on E1E2 antigens derived from a single virus or a combination of antigens selected to maximize genetic diversity. Unfortunately, these vaccines have failed to elicit high bNAb titers (39–42). Therefore, there is a need for selection or design of more effective immunogens. We screened E1E2 proteins from HCV-infected individuals to identify possible antigenic differences between them and discovered several that were sensitive to binding of reference mAbs targeting a diverse array of E2 epitopes. We found that the number of infections with viruses harboring these pan-sensitive E1E2 proteins was highly associated with greater plasma-neutralizing breadth and potency. Notably, E1E2 proteins in this pan-sensitive antigenic cluster were sensitive to the immunodominant bNAbs we identified in broadly neutralizing plasma of reinfected subjects as well as germline precursors of 2 of those bNAbs. We speculate that highly conserved epitopes in bNAb-sensitive, antigenic clade 1 E1E2 proteins are more accessible to Ab binding, favoring selection of B cells expressing bNAb germline precursors. Repeated exposure to heterologous antigenic clade 1 E1E2 variants exposing the same conserved epitope could favor further maturation of bNAb-expressing B cell lineages, while simultaneously disfavoring maturation of B cells targeting less conserved epitopes, which vary across heterologous viruses. On the other hand, bNAb-resistant E1E2 variants (e.g., antigenic clade 4) may occlude conserved epitopes more effectively, favoring selection of B cells targeting more exposed, variable epitopes. This observation could help define characteristics of antigens that should be included in an HCV vaccine.

A desirable vaccine will need to generate an immune response capable of neutralizing very diverse viruses across multiple genotypes. However, whether the viral genotype or genetic distance between infecting strains has significant influence on the development of such a broad response was previously unclear. Here, we showed that greater genetic distance between infecting viruses was not associated with greater neutralizing breadth or potency and that reinfection with an HCV subtype different from the primary infection did not broaden the NAb response. Instead, our data point to repeated exposure to antigenically related, neutralization-sensitive viruses as a better stimulus for bNAb induction. It is interesting to note that both Ab-sensitive and resistant antigenic clades included viruses from multiple genotypes, further indicating that genotypes do not dictate the antigenic characteristics of E1E2 proteins (33).

In agreement with prior studies, greater neutralizing breadth of the plasma Ab response was associated with longer duration of infection and with reinfection (19, 27–29). Notably, in this study, we measured Ab breadth and potency in plasma from multiple reinfections from the same subjects, including subjects who cleared as many as 5 distinct infections. Additionally, all plasma samples were time matched based on DOV between the reinfection and persistence subjects. This approach allowed us to consider number of infections, duration of infection, and outcome of infection as separate variables in the model of bNAb induction. Finally, we identified the anti-HCV bNAb types induced in plasma. By the second or later infection, we observed a focusing of the immune response toward HEPC146-, AR4A-, HEPC74-, and HEPC108-like responses (all broadly neutralizing). It is notable that AR4A- and HEPC74-like responses in plasma have been previously associated with clearance of primary infection (34). AR4A was recently shown to bind to the stalk of E2, while HEPC74 binds to the front layer/CD81-binding site of E2 (35, 43). The 2 other immunodominant plasma bNAbs, HEPC146 and HEPC108, were described more recently (31). HEPC146 binding is focused at the CD81-binding loop of E2. The binding epitope of HEPC108 is less well defined, but appears to include residues in both the central β sheet and the front layer of E2. Although the reference mAbs in the panel include mAbs targeting several distinct antigenic sites, it is likely that the epitopes and function of NAbs developing in different individuals do not exactly match the reference mAbs to which they are most closely related. Therefore, more work is needed to isolate additional mAbs from B cells of HCV-infected or vaccinated subjects and to characterize the neutralizing epitopes critical to bNAb induction.

Despite prior studies showing an association between early plasma-neutralizing breadth and clearance of primary infection, we did not observe a significant association in this study between plasma-neutralizing breadth or potency and outcome of infection (clearance versus persistence) (19, 20, 34). We believe this conclusion to be limited by the small sample size of the clearance group (*n =* 8), as there was a trend toward greater neutralizing breadth in cleared infections. Although induction of bNAbs is the goal for prophylactic vaccine development, clearance of established infection is dictated to a greater extent by neutralization of autologous viruses. Further work is needed to fully understand mechanisms of repeated control of HCV reinfections.

While we observed a significant association of neutralizing breadth and potency with repeated antigenic clade 1 exposure, it is worth noting that antigenic clade 3 E1E2 proteins were also sensitive to binding of most of the reference mAbs. The lack of association between number of antigenic clade 3 infections and breadth may have been dictated by the fact that this clade only included 3 E1E2 proteins, one of which was bole1a. Therefore, clade 3 E1E2 proteins might also be considered as possible vaccine antigens capable of eliciting a broad immune response.

In conclusion, we have identified key features of the stimuli associated with the induction of potent anti-HCV bNAbs in humans. Data here indicate that longer duration of viremia and a greater number of infections are associated with greater plasma-neutralizing breadth and potency. This broadening of the Ab response can be attributed to induction of 4 specific bNAb types that we identified in the plasma upon reinfection. Repeated infection with antigenically related, Ab-sensitive HCV strains was strongly associated with bNAb induction, while genetic distance between primary and reinfecting strains was less important. This study indicates that a prime-boost vaccine strategy with genetically distinct but antigenically similar bNAb-sensitive E1E2 proteins, such as those in antigenic clades 1 and 3, should be considered as a vaccine strategy for inducing potent bNAbs in humans.

## Methods

### Human subjects.

Plasma was obtained from the BBAASH cohort (44). None of the patients in the study were treated for HCV. Plasma samples from persistently infected subjects were time matched with clearance subjects based on DOV. DOV was calculated by only counting viremic periods and excluding the period of aviremia between infections. The first viremic time point and the aviremic time point prior to reinfections were not time matched and therefore were not included in comparisons between groups.

### Cell lines.

HEK293T/17 cells (sex: female) were obtained from ATCC (catalog CRL-11268), maintained in DMEM, and supplemented with sodium pyruvate, 10% heat-inactivated fetal bovine serum, and glutamine. HEP3B cells (sex: male) were obtained from ATCC (catalog HB-8064) and maintained in modified eagle medium supplemented with sodium pyruvate, 10% heat-inactivated fetal bovine serum, nonessential amino acids, penicillin-streptomycin, and glutamine. Cells were cultured at 37°C in a humidified incubator with 5% CO_2_, and monolayers were disrupted at 80% to 100% confluence with Trypsin-EDTA.

### Source of reference bNAbs.

HEPC74, HEPC98, HEPC112, HEPC146, HEPC108, HEPC74rua, HEPC108rua, HEPC146rua, and HEPC98rua were isolated in house (30). mAbs CBH-2, CBH-7, HC-1 (15), HC84.26 (45), and HC33.4 (46) were a gift from Steven Foung (Stanford University School of Medicine, Palo Alto, California, USA). mAbs AR1A, AR3A (11), and AR4A (13) were a gift from Mansun Law (Scripps Research Institute, La Jolla, California, USA).

### HCV viral load and serology testing.

HCV viral loads (IU/mL) were quantified after RNA extraction from serum using a QIAGEN MinElute Virus Column with the use of commercial real-time reagents (Abbot HCV Real-Time Assay) migrated onto a research-based real-time PCR (RT-PCR) platform (Roche 480 LightCycler). HCV seropositivity was determined using the Ortho HCV ELISA Test System, version 3.0 (Ortho Clinical Diagnostics). This assay has a lower limit of detection of 50 IU/mL.

### Viral sequencing.

Total RNA was extracted from plasma using a QIAamp viral RNA Mini Column (QIAGEN), and direct sequencing of reverse-transcription PCR products from the Core-E1 region was performed as described previously (21, 47, 48). Alignment to reference sequences was used to assign HCV subtypes. E1 sequences (H77 nt 943–1288) were used for genetic clustering to identify closely related sequences. Genetic nucleotide distance greater than 0.03 was used to define distinct viral infections and was calculated with HIV-TRACE (49).

### HCVpp production, infectivity, and neutralization assays.

HCVpp were produced by lipofectamine-mediated transfection of HCV E1E2, pNL4-3.Luc.R-E-, and pAdVantage (Promega) plasmids into HEK293T cells as previously described (50). For infectivity testing, HCVpp were incubated on Hep3B target cells for 5 hours before media was removed. The panel of 19 heterologous genotype 1 HCVpp has been described previously (19, 51). All HCVpp used in neutralization assays produced RLU values at least 10-fold above background entry by mock pseudoparticles. Only HCVpp preparations producing at least 1 × 10^6^ RLU were used for neutralization experiments, and HCVpp input was normalized to a maximum of 10 × 10^6^ RLU. We tested each plasma sample in duplicate to control for plate-to-plate variability and in 2 independent experiments using different stocks of panel of 19 HCVpp to control for batch-to-batch variability.

Neutralization assays were performed as described previously (52). HCVpp were incubated for 1 hour with heat-inactivated plasma at a 1:100 dilution or mAb at 10 μg/mL and then added in duplicate to Hep3B target cells for 5 hours before medium was changed. Nonspecific human IgG (Sigma-Aldrich) at 100 μg/mL and heat-inactivated preimmune plasma (PIP) at 1:100 were used as negative control. HCVpp entry was determined after 72 hours by measurement of luciferase activity of cell lysates in RLU. Percentage of neutralization was calculated by the following formula: 1 – (RLU_autologousplasma_/RLU_autologousPIP_) × 100. Based on independent negative control neutralization assays performed in duplicate with isotype control mAb R04 at 100 mcg/mL and 65 different HCVpp (33), nonspecific neutralization in this assay was an average of 1.0%, with a standard deviation of 19.9%. Therefore, we set the cutoff for true-positive neutralization in this assay at less than 25%. Percentages of neutralization values were converted to neutralization profiles for each plasma sample (rank order of HCVpp neutralization with the most sensitive HCVpp ranked 1 and the least sensitive HCVpp ranked 19) for input into the deconvolution algorithm.

### Deconvolution of mAb types in plasma.

Plasma neutralization profiles (rank order of sensitivity of 19 HCVpp to each sample) were averaged across the 2 independent experiments to generate a final neutralization profile for each plasma sample. Neutralization profiles of each plasma were compared with neutralization profiles of a panel of 11 reference HCV-specific mAbs. Neutralization profiles of each of the 3 new reference mAbs added for this study and the 8 reference mAbs selected in a previous study were averaged across 5 independent experiments (34). Deconvolution analysis was performed using Pearson’s correlation between control mAb spike-in experiment or plasma neutralization profiles and neutralization profiles of the 11 reference mAbs to delineate the relative proportion of each reference mAb type present in each test sample, as previously described (34), using code written in R (GitHub repository: https://github.com/BaileyLabHCV/Neutralizing-breadth.git Commit ID: 1420dfb). The true positive deconvolution value for each reference mAb was set based on control mAb spike-in experiments with single mAbs or combinations of 2 or 3 mAbs (Supplemental Table 1). HEPC74 served as a positive control in each plasma-neutralization experiment. We only included plasma neutralization results from experiments where HEPC74 had a breadth of 18 (±1 standard deviation) and where deconvolution of the neutralization profile of HEPC74 obtained in that experiment resulted in a HEPC74-positive result (i.e., proportion of response attributed to HEPC74 surpassed the true positive cutoff (Supplemental Figure 3).

### 5′ Hemigenome SGA and E1E2 cloning.

HCV 5′ hemigenomes from plasma virus were amplified by RT-PCR after limiting dilution to ensure SGA, using previously described methods (53). Hemigenomes from the earliest viremic and last viremic time points for each infection were preferentially selected over sequences from intermediate time points. At each time point, the most frequently observed sequence from a dominant clade (clade with most sequences) was selected. If no sequence was most abundant in the dominant clade, the sequence closest to the MRCA for that clade was selected. In infections with multiple viremic time points, when possible, hemigenomes from the same phylogenetic lineage spanning multiple time points were selected. T/F viruses were identified with clustering by average mutation method of SGA sequences from the earliest viremic time point of an infection. When more than one T/F virus was identified, the most abundant virus was selected based on detection of multiple identical sequences (54). E1E2 genes were PCR amplified from SGA hemigenomes of interest and cloned as previously described (19). Sequences of all E1E2 clones were confirmed after cloning. All original sequence data were deposited in GenBank.

### HCV E1E2 ELISA.

mAb binding to E1E2 was quantitated using ELISA as previously described (30). Briefly, 293T cells were transfected with E1E2 expression plasmids. Cell lysates were harvested at 48 hours. Plates were coated with 500 ng *Galanthus nivalis* lectin (EY Labs) and blocked with PBS containing 0.5% Tween-20, 1% nonfat dry milk, and 1% goat serum; E1E2-containing cell lysates were added. For titration binding curves, mAbs were assayed in duplicate at 3-fold serial dilutions, starting at 10 μg/mL, and binding was detected with HRP-conjugated anti-human IgG secondary Abs (Vector Laboratories PI-3000). Relative protein concentrations in each E1E2 lysate preparation were determined by measuring binding of serial dilutions of control mAb HCV-1, which was selected because the linear HCV-1 epitope was intact in all E1E2 proteins (55). For antigenic profiling, rua mAbs were assayed in duplicate at 10 μg/mL, while the rest of the mAbs were assayed at 0.08 μg/mL. HCV-1 and nonspecific human IgG (Sigma-Aldrich) were included in the antigenic profiling experiment as positive and negative controls, respectively. All lysates were tested against all mAbs in the panel in the same experiment.

### Antigenic profiling data cleanup and clustering.

To exclude ELISA data arising from inconsistent replicate wells, we calculated the absolute difference between OD values of replicate wells measuring nonspecific human IgG binding to each E1E2 protein. We then discarded any OD pairs with absolute difference greater than this average IgG replicate difference plus 2 standard deviations. This led to 4 replicate pairs being deleted for AR3A binding. To remove from the analysis any E1E2 lysates with inadequate protein expression, we calculated the true positive OD cutoff (nonspecific IgG average OD plus 2 standard deviations) and then excluded E1E2 lysates with HCV-1 binding below this true positive cutoff. This led to exclusion of 6 E1E2 lysates from our analysis. HEPC146rua and HEPC98rua did not have detectable binding to any E1E2 proteins, so they were excluded from subsequent analyses. To account for differences in E1E2 protein concentrations, we normalized average OD values for each mAb to the HCV-1 average OD for the same lysate. The resulting OD values were entered into a code in R that clustered E1E2 proteins based on mean squared distance between binding profiles (56), using Ward’s minimum variance method in the hclust R package (56).

### Statistics.

Statistical analyses were performed in Prism (GraphPad software, v7erson.02). Correlations between plasma and reference mAb neutralization profiles were calculated using Pearson’s method. For all comparisons, *P* values of less than 0.05 were considered significant. Breadth and potency models were computed in R. GitHub repository: TIngchangW/Neut-Breadth-Analysis: 2022 with Nicole (GitHub repository: https://github.com/TIngchangW/Neut-Breadth-Analysis.git Commit ID: af99b7f).

### Accession numbers.

The GenBank accession numbers of E1E2 clones expressed are as follows: OK553726–OK554430, OK583165 –OK583829, MZ834892–MZ835192, OK582746–OK583164, OL332220–OL332312, MZ556841–MZ556946, OK502877–OK503334, MZ457964–MZ458098, OK503618–OK504315, OK582292–OK582745. E1E2 sequences included in this study with (GenBank MZ834892–MZ835192) were previously described (16, 30). The Bole1a genomic sequence (GenBank JQ791196) was previously described (36).

### Study approval.

The protocol was approved by the Institutional Review Board of the Johns Hopkins Hospital, and informed consent was obtained from all study participants.

## Author contributions

JRB and ALC conceived the study. MNZ, SW, and GMS performed viral sequencing and sequence analysis. TW and HJ performed statistical analysis. NF and AF performed binding and neutralization experiments. JEC and AIF provided Abs. NF, ALC, SCR, GM, GS, and JRB analyzed the data. NF and JRB wrote the original draft. All authors reviewed and edited the manuscript.

## Supplementary Material

Supplemental data

## Figures and Tables

**Table 1:**
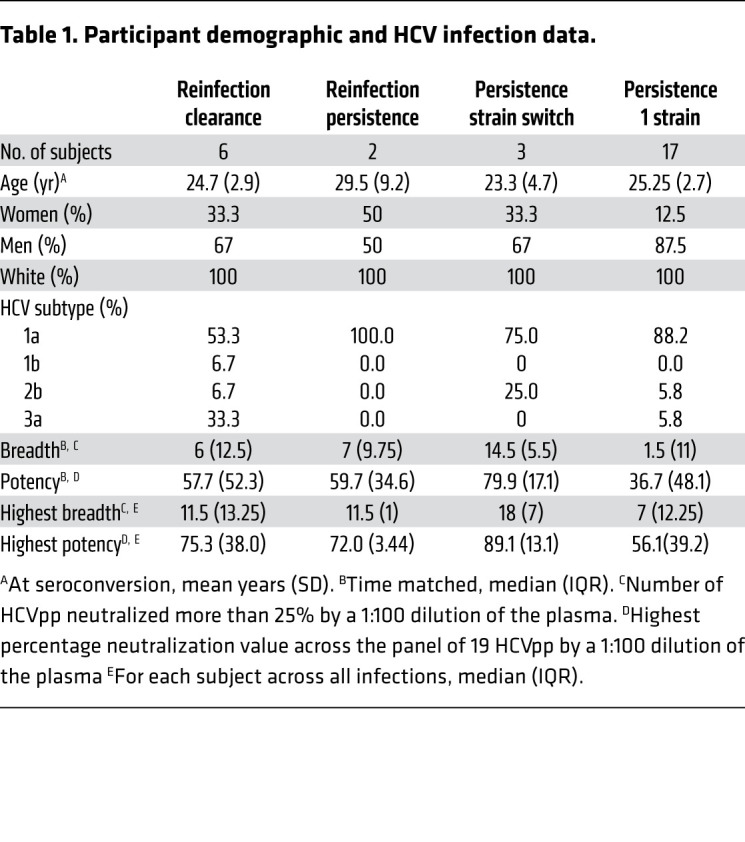
Participant demographic and HCV infection data.

## Version 1 05/19/2022

In-Press Preview

## Version 2 08/01/2022

Electronic publication
