# Supplementary material for: Repeated exposure to heterologous hepatitis C viruses associates with enhanced neutralizing antibody breadth and potency
Source: J Clin Invest. 2022 Aug 1;132(15):e160058. doi: 10.1172/JCI160058 (PMC9337827; doi:10.1172/JCI160058)
Supplement: Supplemental data [file jci-132-160058-s036.pdf]

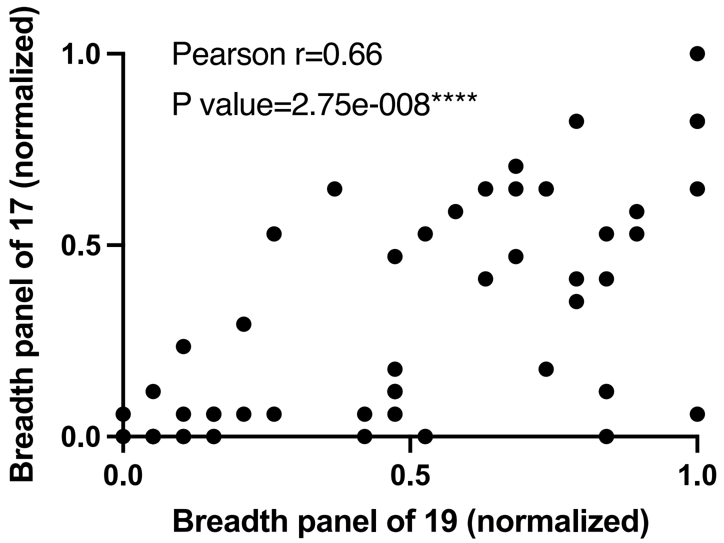

Supplemental Figure 1. Correlation between breadth of participant's plasma (n=57) measured with the panel of 19 genotype 1 HCVpp used in this study, and breadth of the same plasma samples measured with a more recently identified antigenically diverse panel of 17 HCVpp which includes multiple genotypes. Breadth = number of HCVpp neutralized at least 25% by plasma at 1:100 dilution. Breadth for each plasma sample was normalized to account for the number of HCVpp present in each panel. Pearson correlation is shown.

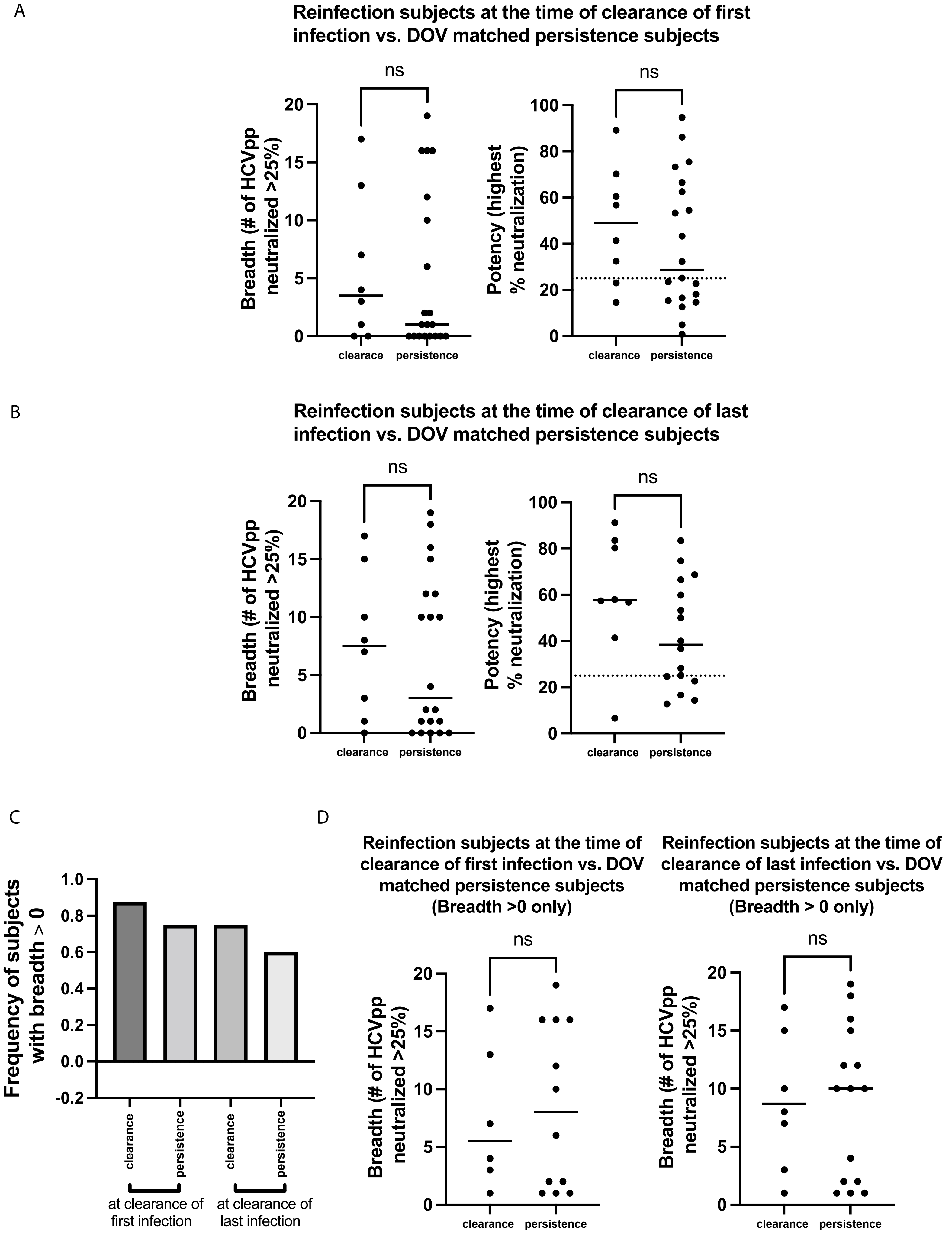

A
Reinfection subjects at the time of clearance of first infection vs. DOV matched persistence subjects
ns
Breadth (# of HCVpp neutralized >25%)
clearace
persistence
Potency (highest % neutralization)
clearance
persistence
B
Reinfection subjects at the time of clearance of last infection vs. DOV matched persistence subjects
ns
Breadth (# of HCVpp neutralized >25%)
clearance
persistence
Potency (highest % neutralization)
C
Frequency of subjects with breadth > 0
clearance
persistence
at clearance of first infection
at clearance of last infection
D
Reinfection subjects at the time of clearance of first infection vs. DOV matched persistence subjects (Breadth >0 only)
Reinfection subjects at the time of clearance of last infection vs. DOV matched persistence subjects (Breadth > 0 only)
ns
Breadth (# of HCVpp neutralized >25%)
clearance
persistence

Supplemental Figure 2. Median plasma neutralizing breadth and potency for clearance samples are not significantly different from time-matched persistence samples. A) Breadth (left) and potency (right) of plasma samples from the time of clearance of primary infection compared to days of viremia-matched plasma samples from persistence subjects. B) Breadth (left) and potency (right) of plasma samples at the time of clearance of their last infection compared to days of viremia-matched plasma samples from persistence subjects. C) Frequency of individuals who developed breadth (breadth greater than 9) in clearance and persistence subjects both at the time of clearance of the first infection and of the last infection. Subjects with breadth lower than 9 were excluded from the analysis. D) Breadth of plasma samples of individuals who developed breadth (breadth greater than 9) from the time of clearance of primary infection (left) and clearance of their last infection (right) compared to days of viremia-matched plasma samples from persistence subjects. Horizontal solid lines indicate medians. Dashed lines indicate 25% neutralization by plasma. Normality of data was tested by Shapiro-Wilk test. Unpaired t test with Welch's correction was run for potency (p-value>0.05) and Mann Whitney nonparametric test was run for breadth (p-value>0.05).

	Reference nAbs (proportion)													
Sample ID	HC84.26	HC-1	CBH-2	AR1A	AR3A	AR4A	HEPC74	HEPC98	HEPC108	HEPC112	HEPC146	Pearson Corr	p-value	Breadth
avg HEPC74 experiment 1	0.00	0.00	0.00	0.00	0.10	0.00	0.83	0.00	0.00	0.07	0.00	0.88	7.07E-07	18
avg HEPC74 experiment 2	0.00	0.00	0.00	0.00	0.09	0.00	0.82	0.00	0.06	0.04	0.00	0.96	2.10E-10	18
avg HEPC74 experiment 3	0.05	0.00	0.12	0.00	0.15	0.00	0.68	0.00	0.00	0.00	0.00	0.92	2.04E-08	18
avg HEPC74 experiment 4	0.00	0.00	0.03	0.00	0.22	0.00	0.75	0.00	0.00	0.00	0.00	0.94	1.31E-09	18
avg HEPC74 experiment 5	0.00	0.00	0.06	0.08	0.17	0.00	0.70	0.00	0.00	0.00	0.00	0.92	3.23E-08	18
avg HEPC74 experiment 6	0.00	0.00	0.01	0.00	0.28	0.00	0.67	0.00	0.00	0.03	0.00	0.95	3.94E-10	18
avg HEPC74 experiment 7	0.17	0.00	0.03	0.00	0.15	0.00	0.54	0.00	0.02	0.08	0.00	0.94	1.24E-09	19
avg HEPC74 experiment 8	0.00	0.00	0.00	0.00	0.05	0.05	0.65	0.02	0.00	0.19	0.02	0.83	9.37E-06	19

Supplemental Figure 3. Consistency of HEPC74 neutralization results across independent experiments was determined by performing deconvolution analysis on each independent experiment. HEPC74 was included in each plasma neutralizing breadth experiment as positive control. Values are the proportion of the neutralization activity of each sample attributed to each reference mAb by the deconvolution analysis. Data from a neutralization experiment was discarded unless HEPC74 displayed neutralizing breadth of 18 (+/- 1 standard deviation) and the HEPC74 deconvolution demonstrated no false positive results and exceeded the true positive cutoff for HEPC74 (0.23).

		Reference nAbs (proportion)													
		Narrow-breadth nAbs					bNAbs								
Subject ID	DOV	AR1A	HC-1	HEPC98	CBH-2	HEPC112*	AR3A	HEPC74*	HC84.26	AR4A	HEPC108*	HEPC146*	Pearson Corr	p-value	Breadth
	D0														0
	D209	0.00	0.00	0.00	0.03	0.00	0.00	0.26	0.00	0.09	0.17	0.43	0.82	0.00	14
C110	D436	0.00	0.02	0.09	0.00	0.00	0.07	0.31	0.00	0.00	0.00	0.51	0.90	0.00	17
	D466	0.00	0.00	0.00	0.00	0.16	0.00	0.34	0.00	0.04	0.11	0.36	0.92	0.00	15
	D1081	0.00	0.00	0.00	0.00	0.04	0.08	0.20	0.00	0.00	0.23	0.44	0.87	0.00	17
	D17														0
	D493	0.00	0.37	0.01	0.00	0.21	0.00	0.00	0.00	0.06	0.00	0.35	0.87	0.00	7
C18	D535	0.00	0.14	0.00	0.00	0.03	0.00	0.00	0.00	0.08	0.24	0.51	0.93	0.00	7
	D863	0.00	0.08	0.00	0.00	0.25	0.00	0.00	0.00	0.17	0.10	0.41	0.92	0.00	8
	D2243	0.00	0.00	0.00	0.00	0.21	0.00	0.00	0.00	0.05	0.34	0.40	0.90	0.00	12

Supplemental Figure 4. Concordance between plasma deconvolution and mAbs isolated from B cells of the same subject. Values are proportion of the neutralizing activity of each plasma sample attributed to each reference mAb by the deconvolution analysis. Values that exceed the deconvolution true positive threshold are underlined. We isolated mAbs from two study subjects (C110 and C18) which are concordant with the deconvolution results for these subjects. HEPC74 was isolated from C110's B cells. HEPC108, HEPC146, and HEPC112 were isolated from C18's B cells (Bailey et al., 2017; Colbert et al., 2019).

A

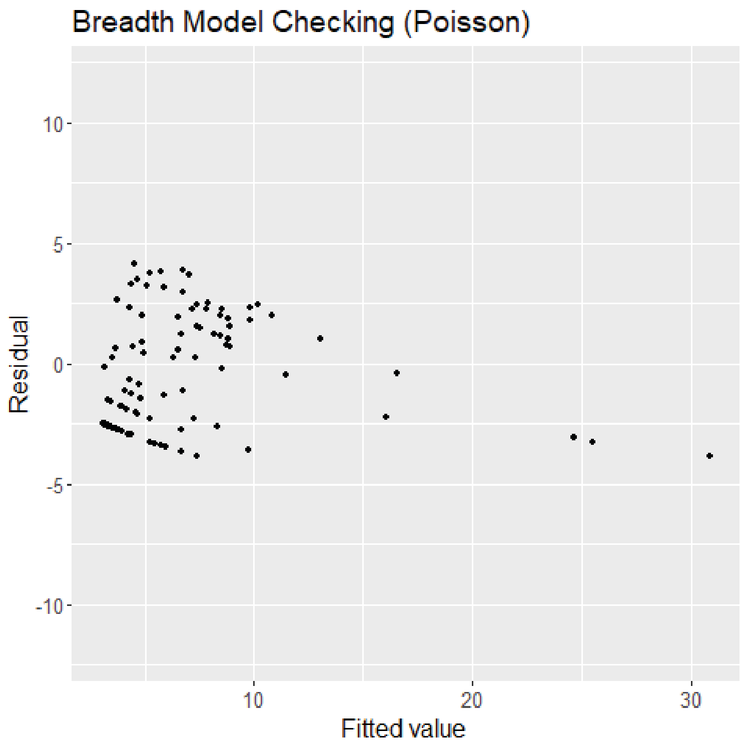

log(E[breadth|number of infections, days of viremia]) =
$\beta o + \beta 1$ *number of infections+ $\beta 2$ * days of viremia

B

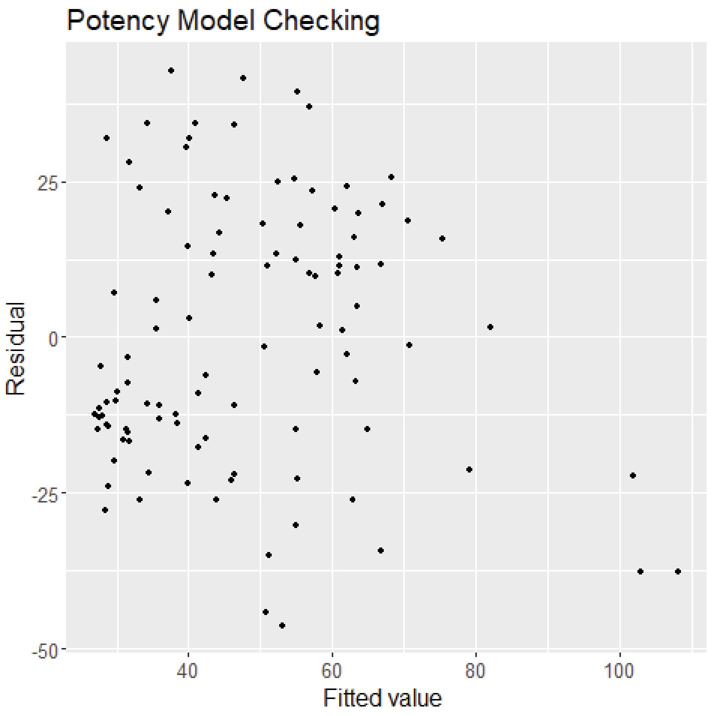

E[potency|number of infections, days of viremia] =
β o + β 1 *number of infections+ $\beta 2$ * days of viremia

Supplemental Figure 5. Model checking. Breadth model checking for the quasi-Poisson regression model (A) and potency model checking for linear regression model (B) were computed in R. The residuals are scattered and well spread. There are no obvious trends for concentration of the residuals. Therefore, the models fit the data well. Equations are shown (below).

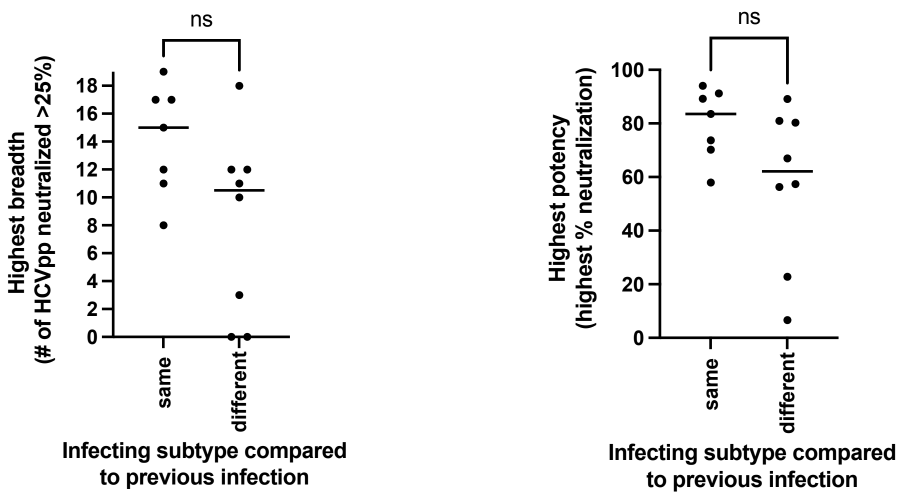

Supplemental Figure 6. No difference in neutralizing breadth or potency in participants reinfected with a virus from the same or different subtype relative to primary infection virus. Mann Whitney nonparametric tests for significance were conducted (p-value>0.05). Horizontal lines indicate medians.

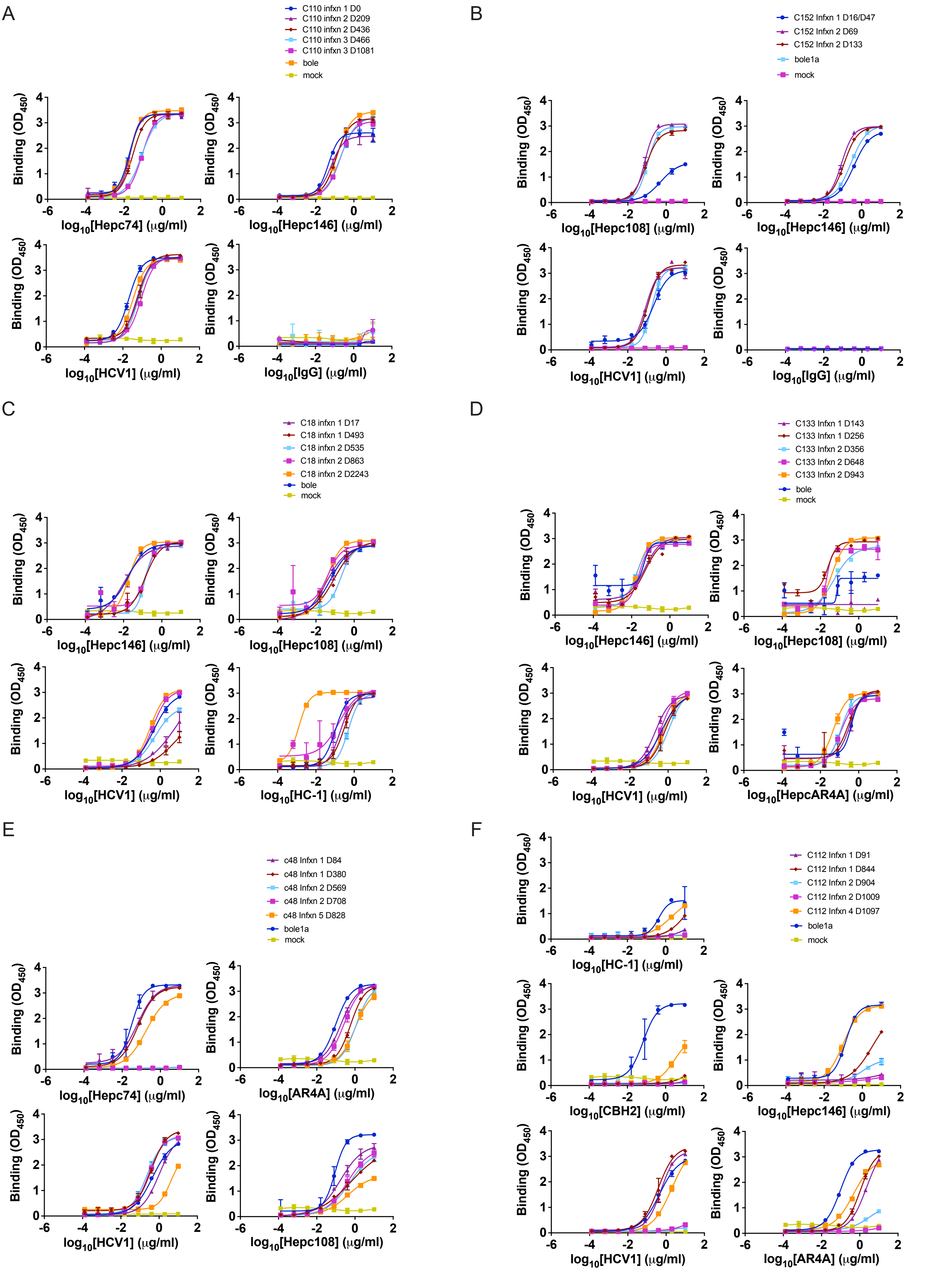

Supplemental Figure 7. Titration binding curves of reference mAbs with E1E2 lysates. Binding of reference mAbs detected by deconvolution analysis in the plasma of reinfection subjects C110 (A), C152 (B), C18 (C), C133 (D), C48 (E), and C112 (F) to longitudinal E1E2 lysates from the same subjects in an ELISA. Each binding measurement was obtained in duplicate and averaged. Error bars indicate standard deviations.

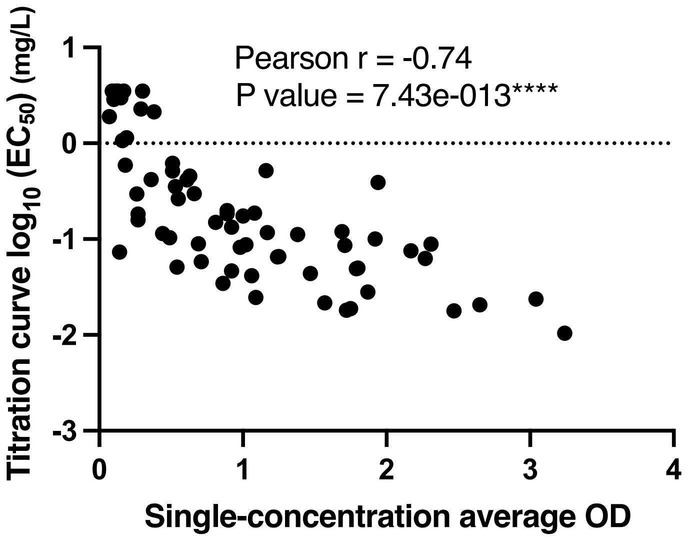

Supplemental Figure 8. Titration curve EC50 of reference mAb binding to longitudinal E1E2 lysates correlate significantly with normalized OD results testing of the same mAb-E1E2 combinations at a single mAb concentration. Pearson correlation is shown. Each mAb titration binding measurement was obtained in duplicate and averaged (Figure S7). Each single mAb concentration binding is the average of 2 OD measurements in an ELISA and is normalized by HCV-1 binding (Figure 6a).

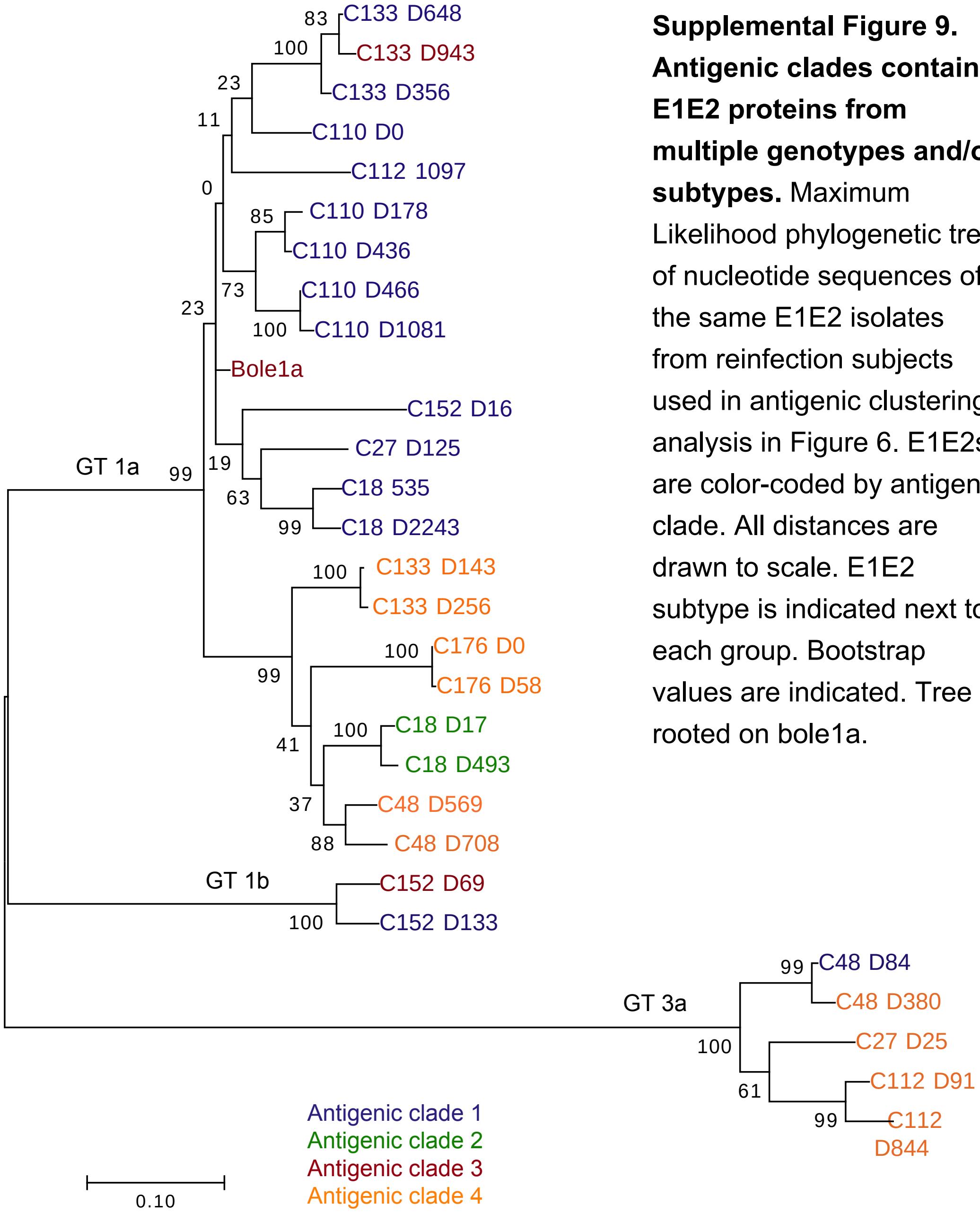

Supplemental Figure 9. Antigenic clades contain E1E2 proteins from multiple genotypes and/or subtypes. Maximum Likelihood phylogenetic tree of nucleotide sequences of the same E1E2 isolates from reinfection subjects used in antigenic clustering analysis in Figure 6. E1E2s are color-coded by antigenic clade. All distances are drawn to scale. E1E2 subtype is indicated next to each group. Bootstrap values are indicated. Tree is rooted on bole1a.

A

Group	Subject	Infxn # (gt)	D.O.V.	# of infxns with clade 1 viruses	# of infxns with clade 2 viruses	# of infxns with clade 3 viruses	# of infxns with clade 4 viruses	# of infxns with distinct clade viruses	Breadth	Potency
Reinfect Clear	C110	1 (1a)	0	1	0	0	0	1	0	0
		2 (1a)	209	2	0	0	0	1	14	72
			436	2	0	0	0	1	16	89
		3 (1a)	466	3	0	0	0	1	14	80
			1081	3	0	0	0	1	16	91
	C112	1 (3a)	91	0	0	0	1	1	1	21
			844	0	0	0	1	1	5	33
		4 (1a)	1097	1	0	0	1	2	15	83
	C48	1 (3a)	84	1	0	0	0	1	1	19
			380	1	0	0	1	2	12	70
		2 (1a)	569	1	0	0	2	2	8	65
			708	1	0	0	2	2	12	67
	C152	1 (1a)	16	1	0	0	0	1	0	16
			47	1	0	0	0	1	4	60
		2 (1b)	69	1	0	1	0	2	3	37
			133	2	0	1	0	2	10	80
	C27	1 (3a)	25	0	0	0	1	1	0	23
		2 (1a)	125	1	0	0	1	2	4	57
	C176	1 (1a)	0	0	0	0	1	1	0	15
			58	0	0	0	1	1	1	15
Reinfect persist	C133	1 (1a)	143	0	0	0	1	1	1	15
			256	0	0	0	1	1	2	41
		3 (1a)	356	1	0	0	1	2	1	16
			648	1	0	0	1	2	11	74
			943	1	0	1	1	3	11	69
	C18	1 (1a)	17	0	1	0	0	1	0	15
			493	0	1	0	0	1	8	57
		2 (1a)	535	1	1	0	0	2	8	62
			2243	1	1	0	0	2	11	70

B

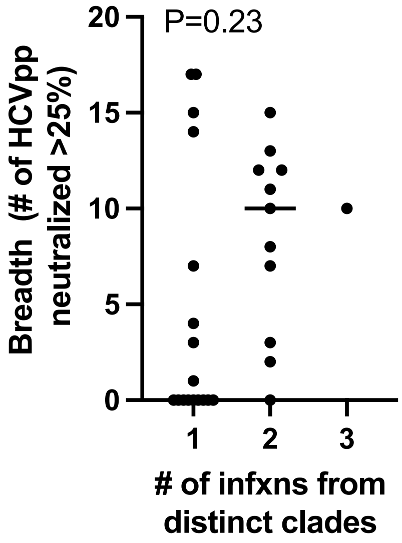

Supplemental Figure 10. Number of infections with viruses from antigenic clade 1-4 are significantly correlated with neutralizing breadth. A) Table summarizing the number of infections with viruses from antigenic clusters 1-4 and from distinct antigenic clusters for reinfection subjects, and the neutralizing breadth of plasma at the same timepoints. B) Number of infections with viruses from distinct clades is not associated with greater neutralizing breadth. One-way ANOVA was conducted (p-value>0.05). Horizontal lines indicate medians.

	Reference mAbs (proportion)										
mAbs added	HC84.26	HC-1	CBH-2	AR1A	AR3A	AR4A	HEPC74	HEPC98	HEPC108	HEPC112	HEPC146
AR4A 1 2	0.00	0.00	0.00	0.00	0.00	0.92	0.08	0.00	0.00	0.00	0.00
AR4A 3 4	0.00	0.00	0.00	0.00	0.00	0.73	0.00	0.00	0.27	0.00	0.00
AR4A 2 3	0.14	0.00	0.00	0.04	0.00	0.76	0.06	0.00	0.00	0.00	0.00
AR4A 4 5	0.00	0.00	0.00	0.09	0.06	0.85	0.00	0.00	0.00	0.00	0.00
AR4A 1 3	0.00	0.00	0.00	0.00	0.00	0.63	0.00	0.00	0.26	0.00	0.11
AR4A 1 4	0.00	0.00	0.00	0.00	0.00	0.78	0.00	0.00	0.17	0.00	0.06
AR4A 1 5	0.00	0.00	0.00	0.00	0.00	0.83	0.00	0.11	0.00	0.06	0.00
AR4A 2 4	0.11	0.00	0.00	0.00	0.00	0.81	0.08	0.00	0.00	0.00	0.00
AR4A 2 5	0.00	0.00	0.00	0.21	0.02	0.61	0.16	0.00	0.00	0.00	0.00
AR4A 3 5	0.00	0.00	0.00	0.12	0.10	0.77	0.00	0.00	0.00	0.00	0.00
AR3A 1 2	0.00	0.00	0.09	0.00	0.71	0.00	0.13	0.07	0.00	0.00	0.00
AR3A 2 3	0.12	0.00	0.17	0.00	0.71	0.00	0.00	0.00	0.00	0.00	0.00
AR3A 3 4	0.00	0.01	0.15	0.00	0.81	0.00	0.00	0.00	0.04	0.00	0.00
AR3A 4 5	0.00	0.02	0.00	0.00	0.72	0.08	0.00	0.00	0.00	0.18	0.00
AR3A 3 5	0.01	0.07	0.00	0.00	0.78	0.06	0.00	0.00	0.08	0.00	0.00
AR3A 1 3	0.00	0.00	0.10	0.00	0.64	0.00	0.20	0.00	0.00	0.00	0.06
AR3A 1 4	0.00	0.00	0.00	0.00	0.81	0.00	0.07	0.01	0.00	0.10	0.01
AR3A 1 5	0.00	0.00	0.00	0.00	0.60	0.15	0.00	0.12	0.00	0.02	0.11
AR3A 2 4	0.00	0.00	0.15	0.00	0.85	0.00	0.00	0.00	0.00	0.00	0.00
AR3A 2 5	0.01	0.00	0.00	0.03	0.85	0.06	0.00	0.04	0.00	0.00	0.00
AR1A 1 2	0.00	0.00	0.00	0.62	0.02	0.07	0.12	0.00	0.00	0.17	0.00
AR1A 2 3	0.00	0.14	0.00	0.75	0.00	0.11	0.00	0.00	0.00	0.00	0.00
AR1A 3 4	0.00	0.00	0.00	0.59	0.00	0.00	0.00	0.01	0.00	0.00	0.40
AR1A 4 5	0.00	0.00	0.00	0.62	0.00	0.00	0.00	0.09	0.00	0.22	0.08
AR1A 1 3	0.00	0.00	0.00	0.61	0.00	0.00	0.00	0.05	0.00	0.00	0.34
AR1A 1 4	0.00	0.00	0.00	0.76	0.00	0.00	0.00	0.07	0.00	0.00	0.17
AR1A 1 5	0.00	0.00	0.00	0.66	0.00	0.00	0.00	0.09	0.00	0.25	0.00
AR1A 2 4	0.05	0.01	0.00	0.89	0.00	0.04	0.00	0.00	0.00	0.01	0.00
AR1A 2 5	0.05	0.00	0.00	0.41	0.00	0.08	0.00	0.00	0.00	0.46	0.00
AR1A 3 5	0.00	0.00	0.00	0.53	0.00	0.00	0.00	0.07	0.00	0.16	0.24
HC84.26 1 2	0.81	0.03	0.00	0.00	0.00	0.07	0.08	0.00	0.02	0.00	0.00
HC84.26 2 3	0.81	0.00	0.00	0.00	0.15	0.00	0.04	0.00	0.00	0.00	0.00
HC84.26 3 4	0.92	0.00	0.00	0.01	0.01	0.00	0.00	0.00	0.06	0.00	0.00
HC84.26 4 5	0.95	0.00	0.05	0.00	0.00	0.00	0.00	0.00	0.00	0.00	0.00
HC84.26 1 3	0.82	0.00	0.00	0.00	0.00	0.00	0.05	0.00	0.13	0.00	0.00
HC84.26 1 4	0.89	0.01	0.00	0.00	0.00	0.10	0.00	0.00	0.00	0.00	0.00
HC84.26 1 5	0.93	0.00	0.06	0.00	0.00	0.00	0.00	0.00	0.00	0.00	0.01
HC84.26 2 4	0.92	0.00	0.00	0.00	0.00	0.05	0.00	0.03	0.00	0.00	0.00
HC84.26 2 5	0.91	0.00	0.06	0.00	0.00	0.00	0.00	0.03	0.00	0.00	0.00
HC84.26 3 5	0.85	0.00	0.15	0.00	0.01	0.00	0.00	0.00	0.00	0.00	0.00
HC-1 1 2	0.00	0.79	0.00	0.00	0.00	0.00	0.00	0.08	0.00	0.06	0.07
HC-1 2 3	0.00	0.76	0.00	0.06	0.07	0.00	0.07	0.00	0.00	0.05	0.00

HC-1 3 4	0.00	0.80	0.00	0.00	0.05	0.00	0.00	0.00	0.15	0.00	0.00
HC-1 4 5	0.01	0.86	0.00	0.05	0.00	0.00	0.00	0.00	0.00	0.08	0.00
HC-1 1 3	0.00	0.75	0.01	0.00	0.00	0.00	0.00	0.04	0.13	0.00	0.07
HC-1 1 4	0.06	0.82	0.00	0.00	0.00	0.00	0.00	0.08	0.00	0.00	0.04
HC-1 1 5	0.00	0.78	0.00	0.00	0.00	0.00	0.00	0.08	0.00	0.02	0.12
HC-1 2 4	0.00	0.83	0.00	0.00	0.07	0.00	0.00	0.00	0.00	0.10	0.00
HC-1 2 5	0.00	0.68	0.00	0.00	0.00	0.01	0.00	0.00	0.00	0.31	0.00
HC-1 3 5	0.02	0.89	0.00	0.09	0.00	0.00	0.00	0.00	0.00	0.00	0.00
HEPC74 1 2	0.00	0.00	0.00	0.00	0.15	0.00	0.72	0.09	0.00	0.00	0.04
HEPC74 2 3	0.00	0.00	0.00	0.00	0.09	0.03	0.66	0.00	0.23	0.00	0.00
HEPC74 3 4	0.35	0.00	0.00	0.00	0.00	0.03	0.62	0.00	0.00	0.00	0.00
HEPC74 4 5	0.13	0.00	0.00	0.00	0.00	0.07	0.80	0.00	0.00	0.00	0.00
HEPC74 1 3	0.08	0.00	0.17	0.00	0.00	0.05	0.55	0.00	0.13	0.00	0.01
HEPC74 1 4	0.01	0.00	0.20	0.00	0.00	0.15	0.64	0.01	0.00	0.00	0.00
HEPC74 1 5	0.00	0.00	0.13	0.00	0.00	0.00	0.78	0.09	0.00	0.00	0.00
HEPC74 2 4	0.00	0.04	0.00	0.00	0.08	0.16	0.72	0.00	0.00	0.00	0.00
HEPC74 2 5	0.00	0.09	0.00	0.00	0.08	0.06	0.77	0.00	0.00	0.00	0.00
HEPC74 3 5	0.17	0.00	0.00	0.00	0.00	0.00	0.76	0.00	0.07	0.00	0.00
HEPC98 1 2	0.00	0.00	0.16	0.00	0.03	0.00	0.00	0.79	0.00	0.02	0.00
HEPC98 2 3	0.00	0.00	0.09	0.01	0.00	0.00	0.00	0.89	0.00	0.00	0.00
HEPC98 3 4	0.00	0.00	0.00	0.19	0.00	0.00	0.00	0.81	0.00	0.00	0.00
HEPC98 4 5	0.00	0.20	0.00	0.00	0.00	0.10	0.01	0.69	0.00	0.00	0.00
HEPC98 1 3	0.00	0.00	0.00	0.09	0.04	0.00	0.00	0.85	0.02	0.00	0.00
HEPC98 1 4	0.00	0.00	0.00	0.00	0.04	0.00	0.00	0.91	0.00	0.05	0.00
HEPC98 1 5	0.00	0.15	0.00	0.00	0.00	0.00	0.04	0.79	0.00	0.02	0.00
HEPC98 2 4	0.00	0.00	0.05	0.00	0.00	0.00	0.00	0.95	0.00	0.00	0.00
HEPC98 2 5	0.00	0.06	0.09	0.00	0.00	0.00	0.01	0.83	0.00	0.01	0.00
HEPC98 3 5	0.00	0.13	0.00	0.06	0.00	0.11	0.00	0.70	0.00	0.00	0.00
CBH-2 1 2	0.00	0.00	0.97	0.00	0.00	0.00	0.01	0.01	0.00	0.00	0.00
CBH-2 2 3	0.00	0.00	0.65	0.00	0.14	0.00	0.21	0.00	0.00	0.00	0.00
CBH-2 3 4	0.00	0.05	0.76	0.00	0.05	0.00	0.05	0.00	0.08	0.00	0.00
CBH-2 4 5	0.00	0.00	0.99	0.00	0.00	0.00	0.00	0.00	0.00	0.01	0.00
CBH-2 1 3	0.11	0.00	0.60	0.00	0.27	0.00	0.00	0.02	0.00	0.00	0.00
CBH-2 1 4	0.00	0.00	0.96	0.00	0.00	0.00	0.00	0.00	0.00	0.04	0.00
CBH-2 1 5	0.03	0.00	0.91	0.00	0.00	0.00	0.00	0.00	0.00	0.06	0.00
CBH-2 2 4	0.00	0.00	0.95	0.00	0.00	0.00	0.05	0.00	0.00	0.00	0.00
CBH-2 2 5	0.00	0.00	0.98	0.00	0.00	0.00	0.02	0.00	0.00	0.00	0.00
CBH-2 3 5	0.13	0.00	0.64	0.00	0.22	0.00	0.00	0.00	0.00	0.00	0.00
HEPC108 1 2	0.13	0.00	0.02	0.00	0.00	0.00	0.26	0.00	0.59	0.00	0.00
HEPC108 2 3	0.00	0.10	0.00	0.00	0.00	0.00	0.00	0.00	0.90	0.00	0.00
HEPC108 3 4	0.04	0.00	0.00	0.00	0.00	0.00	0.16	0.03	0.77	0.00	0.00
HEPC108 4 5	0.03	0.00	0.00	0.00	0.09	0.00	0.24	0.00	0.64	0.00	0.00
HEPC108 1 3	0.00	0.12	0.02	0.00	0.06	0.00	0.00	0.00	0.79	0.01	0.00
HEPC108 1 4	0.00	0.11	0.00	0.00	0.14	0.00	0.00	0.00	0.64	0.00	0.11

HEPC108 1 5	0.05	0.07	0.00	0.00	0.21	0.06	0.00	0.00	0.59	0.00	0.02
HEPC108 2 4	0.16	0.00	0.00	0.00	0.00	0.12	0.11	0.00	0.62	0.00	0.00
HEPC108 2 5	0.05	0.00	0.00	0.00	0.00	0.00	0.00	0.00	0.77	0.00	0.18
HEPC108 3 5	0.08	0.00	0.00	0.00	0.00	0.04	0.00	0.00	0.84	0.04	0.00
HEPC112 1 2	0.00	0.04	0.01	0.00	0.00	0.00	0.00	0.00	0.00	0.94	0.00
HEPC112 2 3	0.08	0.03	0.00	0.24	0.00	0.00	0.00	0.00	0.00	0.66	0.00
HEPC112 3 4	0.00	0.00	0.00	0.07	0.00	0.00	0.00	0.00	0.02	0.90	0.00
HEPC112 4 5	0.00	0.00	0.03	0.00	0.02	0.00	0.00	0.00	0.00	0.95	0.00
HEPC112 1 3	0.00	0.02	0.08	0.07	0.00	0.00	0.00	0.00	0.00	0.83	0.00
HEPC112 1 4	0.00	0.00	0.05	0.19	0.00	0.00	0.00	0.00	0.00	0.76	0.00
HEPC112 1 5	0.00	0.00	0.00	0.00	0.04	0.00	0.00	0.04	0.00	0.92	0.00
HEPC112 2 4	0.00	0.03	0.00	0.00	0.00	0.00	0.00	0.04	0.00	0.91	0.02
HEPC112 2 5	0.00	0.01	0.00	0.20	0.00	0.00	0.00	0.08	0.00	0.65	0.07
HEPC112 3 5	0.00	0.16	0.00	0.04	0.00	0.00	0.00	0.03	0.00	0.69	0.08
HEPC146 1 2	0.00	0.17	0.00	0.00	0.00	0.14	0.00	0.00	0.12	0.00	0.57
HEPC146 2 3	0.04	0.20	0.00	0.12	0.00	0.00	0.00	0.00	0.00	0.00	0.64
HEPC146 3 4	0.00	0.00	0.00	0.15	0.00	0.22	0.00	0.00	0.08	0.12	0.43
HEPC146 4 5	0.00	0.00	0.00	0.03	0.09	0.37	0.00	0.00	0.00	0.00	0.51
HEPC146 1 3	0.00	0.19	0.03	0.00	0.09	0.18	0.00	0.00	0.00	0.00	0.52
HEPC146 1 4	0.00	0.00	0.00	0.09	0.00	0.20	0.00	0.00	0.00	0.13	0.59
HEPC146 1 5	0.00	0.00	0.00	0.05	0.22	0.00	0.00	0.03	0.00	0.06	0.64
HEPC146 2 4	0.00	0.02	0.00	0.00	0.00	0.00	0.05	0.00	0.07	0.14	0.72
HEPC146 2 5	0.00	0.00	0.00	0.05	0.00	0.00	0.00	0.06	0.00	0.20	0.69
HEPC146 3 5	0.00	0.27	0.01	0.00	0.00	0.00	0.00	0.13	0.00	0.01	0.58
HEPC146+AR3A+HC84.26	0.63	0.00	0.00	0.00	0.06	0.10	0.05	0.00	0.16	0.00	0.00
HEPC146+HEPC74	0.00	0.00	0.00	0.00	0.00	0.13	0.47	0.00	0.10	0.07	0.23
HEPC112+AR3A+HEPC98	0.00	0.02	0.00	0.00	0.49	0.00	0.00	0.22	0.00	0.26	0.00
HEPC112+AR4A	0.00	0.04	0.00	0.04	0.00	0.58	0.00	0.00	0.12	0.01	0.22
HEPC108+HEPC98+CBH-2	0.00	0.19	0.00	0.00	0.00	0.05	0.02	0.41	0.33	0.00	0.00
HEPC108+AR1A	0.00	0.00	0.00	0.00	0.00	0.34	0.09	0.00	0.52	0.05	0.00
HC84.26+HEPC74+HEPC98	0.35	0.07	0.00	0.00	0.00	0.00	0.32	0.26	0.00	0.00	0.00
AR3A+HC-1	0.00	0.25	0.00	0.00	0.65	0.00	0.00	0.00	0.10	0.01	0.00
AR3A+CBH-2	0.00	0.00	0.11	0.00	0.89	0.00	0.00	0.00	0.00	0.00	0.00
AR3A+HC-1+CBH-2	0.00	0.12	0.20	0.00	0.43	0.00	0.00	0.00	0.14	0.12	0.00
AR1A+AR4A	0.01	0.02	0.00	0.00	0.00	0.81	0.03	0.00	0.13	0.00	0.00
Mean false positive	0.07	0.09	0.08	0.09	0.09	0.11	0.09	0.06	0.11	0.09	0.11
Std Dev false positive	0.07	0.07	0.06	0.07	0.07	0.08	0.07	0.04	0.07	0.10	0.10
Mean + 2 Std Dev (Positive cutoff)	0.22	0.23	0.20	0.22	0.23	0.27	0.23	0.13	0.25	0.29	0.31

Supplemental Table 1. The true positive deconvolution threshold value for each reference mAb was determined with single mAb and mAb combination spike-in experiments. The reference neutralization profile for each mAb was determined by measuring neutralization of the panel of 19 HCVpp in five independent experiments and averaging the neutralization profiles (rank order of sensitivity of 19 HCVpp) across all experiments. Single mAb spike-in ‘test’ data for input into the deconvolution algorithm was generated by averaging the neutralization profiles from every combination of two of the five independent experiments for each mAb in a pairwise manner (10 ‘test’ neutralization profiles for each mAb). These 10 ‘test’ neutralization profiles were then used as input into the deconvolution algorithm. The numbers (1-5) following the mAb name in the ‘mAb added’ column indicate the pair of independent experiments used as input into the deconvolution algorithm. mAb combination ‘test’ neutralization profiles for input into the deconvolution algorithm were generated by averaging the neutralization profiles from two independent neutralization experiments with 2 or 3 mAbs mixed at equal concentrations. Values in gray are the highest deconvolution values for each spike-in experiment. The true positive threshold for each mAb was determined by calculating the mean + 2 standard deviations of all false positive values for each reference mAb across all experiments.

Subject ID	Infection # (gt)	DPI	Number of T/F viruses	Number (%) of sequences identical to the most abundant T/F virus
C112	1 (3a)	D91	3-10	13 (32.5%)
C48	1 (3a)	D84	>10	2 (3.12%)
C152	1 (1a)	D16	<3	16 (45.7%)
C27	1 (3a)	D25	<3	5 (14.7%)
C176	1 (1a)	D0	3-10	23 (50%)
C110	1 (1a)	D0	>10	1 (3.12%)
C18	1 (1a)	D17	3-10	13 (43.3%)
C133	1 (1a)	D143	<3	14 (36.8%)

Supplemental Table 2. Number of primary infection T/F viruses detected in reinfection subjects. The number of T/F viruses was calculated by AvThreshClust (cluster by average mutation method). We use <3,3-10 and >10 to define the results. When more than one T/F virus was identified, the most abundant virus was selected. Sequences amplified in multiple independent reactions were considered identical when the nucleotide p distance equals 0. % of sequences was determined by dividing the number of sequences identical to the most abundant T/F virus by the total number of sequences amplified from the same viremic timepoint.
